# Breaking Bad: Deagglomerating TiO_2_ in 3D Printable Polymer Composites for Photocatalysis in Environmental Media

**DOI:** 10.1021/acsami.5c23498

**Published:** 2026-02-16

**Authors:** Alan J. Kennedy, Arit Das, Christopher Williams, Lucinda Slattery, Stephen Martin, Matthew Hull, Christopher Griggs, Michael J. Bortner

**Affiliations:** † Environmental Laboratory, 57629 US Army Engineer Research and Development Center, Building 3270, 3909 Halls Ferry Rd, Vicksburg, Mississippi 39180, United States; ‡ Macromolecules Innovation Institute, 1757 Virginia Polytechnic Institute and State University, ICTAS II, Suite 130, 1070 Life Science Circle, Blacksburg, Virginia 24061, United States; § Department of Chemical Engineering, Virginia Polytechnic Institute and State University, 635 Prices Fork Road, Goodwin Hall, Suite 245, Blacksburg, Virginia 24061, United States; ∥ Department of Mechanical Engineering, Virginia Polytechnic Institute and State University, 445 Goodwin Hall, 635 Prices Fork Road, Blacksburg, Virginia 24061, United States; ⊥ Geotechnical and Structures Laboratory, US Army Engineer Research and Development Center, Building 6000, 3909 Halls Ferry Rd, Vicksburg, Mississippi 39180, United States; # Institute for Critical Technology and Applied Science, Virginia Polytechnic Institute and State University, 325 Stanger Street, Suite 410, Blacksburg, Virginia 24061, United States

**Keywords:** photocatalysis, nanocomposite, processing, additive manufacturing, compounding, dispersion

## Abstract

The fields of contaminant destruction and polymer nanocomposites are converging to immobilize photocatalysts for the degradation of conventional and emerging contaminants. Novel work exploits the design freedom of high-surface-area structures enabled by Additive Manufacturing to produce customizable, high-surface-area infilled structures containing photocatalysts. While investigations of nanoparticle dispersion in polymers for mechanical performance are available, there remains a specific need for environmental application-focused research to understand how processing impacts nanocomposite structure–property relationships for 3D printing in water treatment applications. This study investigated twin screw extrusion process parameters (temperature, screw speed, and number of extrusions) on the dispersion of photocatalytic TiO_2_ particles in a 3D printable polylactic acid (PLA) composite, the resulting effects on thermal processing properties, and ultimately whether there were benefits to photocatalytic performance. A Design of Experiments evaluated the aforementioned compounding parameters on the number, size, and location of TiO_2_ agglomerates in PLA (≈20% w/w TiO_2_). Significant correlations between different TiO_2_ dispersion states and thermal processing parameters were revealed. Higher TiO_2_ loadings (≈30 w/w) resulted in higher viscosity and modulus and a smaller processing window for reliable 3D printing. However, all printed structures tested demonstrated similar photocatalytic rates (≈0.32 to 0.37 ± 0.02 h^–1^). This is attributable to the observation of better dispersed TiO_2_ at the surfaces of printer extrudates and the actual printed structures, despite the differences in the initial agglomerate state related to larger agglomerates within the interior of the filament. These results suggest that TiO_2_ dispersion and distribution by twin screw extrusion are sufficient to achieve environmentally effective degradation rates if agglomerates are less than approximately 20 μm (an image analysis cutoff) and if these smaller agglomerates remain near the surface of printed structures. When such dispersion states are achieved, additional efforts to break up agglomerates appear nonessential for acceptable photocatalytic performance.

## Graphical abstract

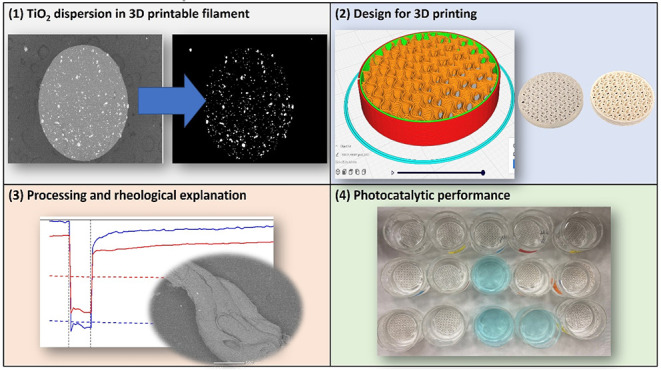

## 1 Introduction

Applying Additive Manufacturing (AM) for structural applications has gained research interest.^1,2^ Similarly, investigating the potential environmental applications of AM using inexpensive and accessible desktop 3D printing (3DP) technologies is increasing.^3^ Examples of AM-enabled environmental application research include novel membranes, bioreactors,^4,5^ passive sampling,^6^ chemical adsorption, and destructive technologies for wastewater treatment (WWT).^3,7–10^ We focus on advanced oxidative processes (AOP), which are conceptually attractive for environmental applications, as they are reusable and self-cleaning, reduce microbial activity, and degrade toxic chemicals into more benign molecules (e.g., H_2_O, CO_2_, N).^11,12^ Mechanisms for contaminant degradation by AOP are well described.^13,14^ Titanium dioxide (TiO_2_) photocatalysts are activated by UV light to produce reactive oxygen species (ROS; OH^•^, ^•^O_2_^–^) that break down toxins (e.g., from harmful algal blooms) into harmless byproducts^14,15^ and are reusable for multiple applications as radicals continue to be generated with irradiation^8,16^ and thus are an environmentally friendly when immobilized; even if released, TiO_2_ has low toxicity, likely from physical effects, when not photoirradiated.^17^

While the body of photocatalysis research for WWT is extensive,^18^ recent review papers^10,19^ have summarized innovative photocatalytic polymer composite applications using 3DP as the technology enabling tool to generate high-surface-area structures^20^ for conventional^8,11,16,21^ and emerging contaminants of environmental concern (e.g., per- and polyfluorinated substances^22,23^ and harmful algal blooms^15,24^ ). The 3DP-AOP process is attractive for environmental applications, since it reduces toxic chemical concentrations without addition of unrecoverable chemicals/oxidizers.^25^ These reviews summarize applications using different 3DP technologies, environments, and WWT applications for various chemicals. However, while dispersion of filler particles in micro- and nanocomposite polymer mechanical strength applications is commonly studied in the structural composite applications literature,^26–28^ less attention is given to polymer composite process controls, filler particle dispersion properties, and their relative importance in environmental applications. Zhu et al.^29^ investigated how compression molding and twin screw extrusion versus thermoforming of relatively low 2% loadings of TiO_2_ in thin polymer films impacted mechanical, barrier, and photocatalytic properties using anionic methylene orange dye.

Research is needed to determine if additional efforts to better disperse TiO_2_ in 3D-printed photocatalytic structures can (1) impact thermal processing parameters and (2) increase chemical treatment efficacy or rates, which is a need discussed for harmful algal bloom toxins as environmental media complexity (e.g., organic matter, ionic strength, light attenuation) increases. The present study, which applies treatment in test waters of environmentally relevant ionic strength, is the first to investigate how the photocatalytic properties of elevated loadings (>20% w/w) of TiO_2_ in 3D-printed composites using the cationic methylene blue dye (as a surrogate for other toxins) are impacted by twin screw processing parameters through Design of Experiments (DoE); this work includes analysis of how rheological recovery and degree of crystallization may impact printing, structure, and TiO_2_ dispersion. The goals build from our previous work^15,30^ reporting photocatalytic efficacy of printable polymer composite photocatalytic structures to understand the importance of process controls in compounding nanoscale TiO_2_ particles. Notably, this study focuses on how varying twin screw process controls for extruding 3D printable polymers impact agglomerate size and location (before and after printing). Nano-TiO_2_ agglomerate size in processed polymers can be substantial at higher loadings (e.g., >8–10% w/w),^12,15^ but the importance of varying states of dispersion of these agglomerates on 3D printing for photocatalytic performance is not well understood. The general composite literature investigates the use of compatibilizers to improve the dispersion and distribution of particles in polymer composites.^26^ However, the research focus herein is on process controls that impact the TiO_2_ agglomerate state in Material Extrusion (MatEx), specifically Fused Filament Fabrication (FFF) extruded filaments and 3D-printed structures, coupled with the associated importance of processing and agglomerate state on photocatalytic performance. This approach avoids the addition of chemical additives, dispersants, stabilizers, or compatibilizers that potentially leach out with time and have adverse environmental implications.^31^ It also avoids additional processing steps and allows the use of off-the-shelf materials. The work here is focused on MatEx-FFF due to its popularity, accessibility, user-friendliness, and low cost.

The three research questions addressed in this work are (1) how twin screw compounding of TiO_2_ with PLA impacted the dispersion state; (2) if these acquired differences in the dispersion state impacted the thermal properties of the nanocomposite; and (3) how these differences in dispersion states may impact or improve photocatalytic performance. We hypothesized that (a) sufficient TiO_2_ loading (≈20% w/w) would have photocatalytic efficacy regardless of the preparation method; (b) extremes in thermal processing would significantly alter agglomerate size; and (c) reduced agglomeration would improve photocatalytic performance. All analyses directly support these questions through a DoE with the intent to make inferences about thermal processing impacts on characterized dispersion and 3D printability of the extruded PLA–TiO_2_ filaments and to determine the photocatalytic rate of different composites relative to their state of TiO_2_ agglomeration. This research informs the development of process-controlled, AM-fabricated photocatalytic structures for environmental applications focused on the destruction of contaminants of concern in surface water and drinking water.

## 2 Materials and Methods

### 2.1 Test Materials

The thermoplastic polymer PLA (SKU: PLA15000, 3DXTECH, Grand Rapids, MI) was selected due to its wide processing window^32^ allowing “printability” with high micro- and nanoparticle loadings^15^ and for its biocompatibility, biodegradability, and sustainability compared to more hydrophobic, hydrocarbon-derived polymers.^33,34^ Nanoscale TiO_2_ (CAS 13463–67–7, 19 nm, SSA 35–65 m^2^/g, Aeroxide P25, Acros Organics. Code: 384290010) was selected for its photocatalytic efficacy from published work.^15,21,22^ We characterized this TiO_2_ as 86% anatase and 14% rutile,^15^ with the anatase form providing its photocatalytic property.^35^ Prior to use, PLA was dried overnight at 50 °C (Fisher Scientific, Isotemp Oven Model 655G), below its 60 °C glass transition temperature (*T*_g_).^36^ The TiO_2_ was dried overnight at 100 °C. Methylene blue (MB) trihydrate (CAS 7220–79–3, CAT#126751000, 95% pure, ACROS Organics, NJ) was used as a widely accepted photocatalytic performance standard.^37^

### 2.2 Polymer–TiO_2_ Compounding

The PLA pellets and TiO_2_ were compounded and extruded by twin screw extrusion (Process 11, Thermo Fisher, 567–7000) through a 2 mm die as a 3D printable filament collected on a filament spooler (ThermoFisher 567–7688). This method uses thermal and mechanical energy to melt-process polymer pellets with conveying and kneading elements to thoroughly mix fillers and extrude the polymer composite filament as a single process relevant to industry and does not require solvents.^38^ An 11 mm single screw feeder (Thermo Fisher No. 567–7656) delivered PLA pellets, and a volumetric MiniTwin feeder (Thermo Fisher No. 567–7660) delivered TiO_2_ to the screw configuration (052–2983d; L/D 40). The feeder speeds were mass per minute calibrated by delivering the material to a tared weigh boat and generating a linear regression. Screw speeds (300 or 500 rpm) and extrusion temperatures (180 or 220 °C) followed a DoE (Table 1). Zones 3–8 were set to temperatures indicated in Table 1, while the hopper and die were 140 and 160 °C, respectively.

**Table 1:** Design of Experiments (DoE) Summary, Using an L8 Taguchi Orthogonal Array (23 Experimental Equivalents)^a^

treatment	screw temperature (°C)	screw speed (rpm)	number extrusions	measured TiO_2_ (% w/w)
TRMT#1	180	300	1×	22%
TRMT#2	180	300	2×	24%
TRMT#3	220	500	1×	20%
TRMT#4	220	500	2×	19%
TRMT#5	180	500	1×	18%^b^
TRMT#6	180	500	2×	19%^b^
TRMT#7	220	300	1×	20%
TRMT#8	220	300	2×	19%

a The temperature profile along the barrel was as indicated, with the temperatures at the hopper and die set to 140 and 160 °C, respectively. Measured TiO_2_ within the tested filaments was determined from thermogravimetric analysis.

b Treatment was also separately prepared at 27% TiO_2_ w/w to determine the impact of a higher loading for 3D printing in viscosity experiments.

### 2.3 Design of Experiments

A Taguchi DoE^39^ was used to efficiently determine the importance of 3 twin screw extrusion processing parameters (barrel temperature, screw speed, and number of extrusions) serving as independent variables, or “factors”, on the extent of TiO_2_ agglomeration in the PLA composite filaments. The DoE was an L8 orthogonal array, and the selected factors and levels of each factor are summarized in Table 1. For example, the number of extrusions was conducted at two levels: one (1×) or two extrusions (2×). The 2× extruded materials used materials produced from their associated 1× treatment (TRMT) pairing (i.e., TRMT#2 was prepared from TRMT#1, TRMT#4 from TRMT#3, TRMT#6 from TRMT#5, and TRMT#8 from TRMT#7) after being repelletized (Varicut Pelletizer 11 mm, Thermo Fisher No. 567–7672) and extruded a second time under the same process parameters.

### 2.4 Polymer Composite Characterization

#### 2.4.1 Size Exclusion Chromatography

The number-averaged (*M*_n_), weight-averaged molecular weight (*M*_w_) and molecular weight distribution (Đ) of the as-received neat PLA pellets and extruded filaments (1×, 2×, and 3×) were characterized using size exclusion chromatography (SEC). While the DoE involved 1× and 2× extrusions, a 3× extrusion was added here to observe trends in molecular weight changes. The samples were dissolved in tetrahydrofuran containing trace amounts of butylated hydroxytoluene as an inhibitor. The dissolved solutions were tested at 1 mL/min and 30 °C using two MIXED-B Agilent PLgel 10 μm columns. The columns were equipped with a Wyatt Optilab Rex refractive index detector (RI) and a Wyatt Dawn Heleos 2 light scattering (LS) detector. The refractive index increment (d*n*/d*c*) value for PLA was 0.042 mL/g.^40^

#### 2.4.2 Particle Analysis

The TiO_2_ size distribution prior to compounding was characterized for volume-intensity-weighted size distribution with a Mastersizer 3000 using software version 3.88 (Malvern Instruments, Cambridge, MA) set to the spherical particle type. Powder was added to reverse osmosis (RO) water until the software indicated adequate laser obscuration (20% max) while being mechanically stirred using a HydroEV at 3000 rpm.

After compounding, cross sections of PLA–TiO_2_ filaments were cut at five randomly selected positions along the length of the extruded filaments using a razor blade, placed on carbon tape, and imaged using a scanning electron microscope (JEOL, JCM-6000PLUS, Peabody, MA) under low vacuum (0.1–0.5 mbar) at 15 kV by a backscattered electron detector. White agglomerates, confirmed to be TiO_2_ by energy dispersive X-ray spectroscopy,^15^ were characterized for total area and total number of agglomerates/mm^2^ in filament cross sections using ImageJ Software (v1.5.4, National Institutes of Health^41^) by a process summarized in Figure S1. SEM images were preprocessed as 8-bit images, and the pixel scale was set to microns (using the SEM scale bar). The image threshold was adjusted (Yen, Black & White), and the dark background was despeckled to reduce pixel noise. The region of interest was indicated as the filament cross section using the circle polygon tool, and the agglomerates were analyzed as defined by the constraints of 0–1 circularity and 10-infinity pixels (i.e., >20 μm diameter). The total percent agglomerate area was determined as the ratio of the TiO_2_ agglomerate area and the area of the filament. The total number of agglomerates was determined from the outline counting feature of the software (Figure S1) and was subsequently standardized to the number per mm^2^ to account for any variation in filament cross-sectional area. This process was also applied to analyze the SEM images of extrudates from the 3D printer (prior to deposition) and the 3D-printed structures that were fully solidified and tested in photocatalysis experiments.

#### 2.4.3 X-ray Microscopy (XRM)

The radial distribution of TiO_2_ within filament cross sections was also characterized by XRM (Zeiss, Xradia Versa 620, White Plains, NY) as a confirmatory analysis to determine where the largest agglomerates were located. The scan results were processed in an ORS Dragonfly Pro from Comet Technologies (Montreal, Canada). The scan was conducted with a 20× objective lens (no source filter) and set to 80 kV voltage and 100 μA current with 2 s exposure time. The objective lens and detector led to a projection pixel size between 0.750 and 0.955 μm. The TiO_2_ was segmented from the PLA and air volume in the scan via density/luminosity thresholding. The TiO_2_ agglomerates were then condensed in the direction of extrusion to produce a radial view of the distribution of the agglomerates relative to the center of mass.

#### 2.4.4 Thermogravimetric Analysis (TGA)

The total mass of TiO_2_ within filaments was determined by TGA (TA Instruments, TA-5500–0510) based on the percentage of sample mass remaining following thermal degradation of all PLA, as previously described.^15^ Briefly, 10–14 mg of samples were placed on platinum pans, and the temperature was ramped from 20 to 600 °C at a rate of 10 °C/min in air. The mass remaining at 600 °C was used to represent the TiO_2_ mass loaded, expressed as a total of the filament dry weight.

#### 2.4.5 Differential Scanning Calorimeter (DSC)

A DSC (TA Instruments, DSC25) was used to determine the glass transition temperature (*T*_g_), peak melting temperature (*T*_m_), and crystallization temperature (*T*_c_) of neat PLA and the PLA–TiO_2_ composites. The protocol involved three segments: first, heat from 20 to 220 °C (10 °C/min), cooling from 220 to 20 °C (5 °C/min), and second, heat from 20 to 220 °C (10 °C/min). The maximum temperature (220 °C) was selected to avoid polymer degradation in the DSC (based on TGA results), which can occur at higher temperatures for PLA^42^ but can be reduced by the presence of TiO_2_.^12^ Since crystalline content in composites can dictate the viscosity and mechanical performance of printed parts,^43^ the degree of crystallinity (*X*_c_) in the PLA–TiO_2_ composites was calculated by eq 1

1 $$d\mathrm{egree}\;\mathrm{of}\;\mathrm{crystallinity}\;\left(\%\right)\;X_{c}=\frac{\Delta H_{m}-\Delta H_{cc}}{\Delta H_{m}^{100}\times \left(1-w_{filler}\right)}\times 100\%$$

where *w*_filler_ is the TiO_2_ weight fraction in the composite filaments, Δ*H*_m_ is the enthalpy of melting, Δ*H*_cc_ is the enthalpy of cold crystallization, and Δ*H*_m_^100^ is the enthalpy of melting of 100% crystalline PLA (Δ*H*_m_^100^ = 93 J/g).^44^

#### 2.4.6 Rheology

A rotational rheometer (DHR-20, TA Instruments) was used to measure the viscosity (η) in shear (0.76 correction accounting for variable rates across the plates^45^), and the complex viscosity (η*), storage modulus (*G*′), and loss modulus (*G*″) were determined in small amplitude oscillatory shear (SAOS). The 25 mm parallel plate geometry was selected as recommended for high viscosity polymer composites with a gap between 10 and 50× larger than the largest particle size dimension.^46^ A gap of 1 mm was selected based on the TiO_2_ primary particle size of 19–25 nm, primary aggregate size ≥70 nm,^47,48^ and measured agglomerate size ranging from 113 to 75,000 nm (Figure S2). The frequency sweep experiments (628 to 0.1 rad/s) used a strain (0.4%) determined to be within the linear viscoelastic region (LVR) based on amplitude sweeps at the low (180 °C) and high (220 °C) temperature extremes of the processing range. All samples (compacted between parallel plates as pellets) were heated to the desired isothermal test temperature (or at least to 180 °C if tested at a lower temperature) and soaked for 180 s prior to data acquisition.

Viscosity data were fit using the Carreau^49^ model (eq 2) if they exhibited a Newtonian Plateau or the Power Law^49^ model (eq 3) when shear thinning was observed throughout the tested range of rates. Shear rates at the wall (γ̇_w_) were determined from eq 4 ^49^

2 $$\eta \left(\mathrm{\gamma ̇}\right)=\eta_{0}{\left[1+{\left(\lambda \mathrm{\gamma ̇}\right)}^{a}\right]}^{n-1/a}$$

3 $$\eta ={K\left|\mathrm{\gamma ̇}\right|}^{n-1}$$

4 $${\mathrm{\gamma ̇}}_{w}=\frac{8\left(3n+1\right)Q}{n{\pi D}^{3}}$$

where η_o_ is the zero shear viscosity, λ is a relaxation time, γ̇ is the shear rate (or frequency), *a* is the Newtonian plateau-shear thinning transition parameter, *n* is the plasticity index, *K* is the power law flow consistency index, *D* is the filament diameter, and *Q* is the volumetric flow rate (nozzle cross-sectional area ● extrudate velocity).

Three interval thixotropy tests (3ITT) were conducted at 200 °C in steady shear to determine viscosity recovery and in SAOS to determine modulus magnitude and recovery after deformations (10%) outside the LVR. The viscosity recovery tests consisted of a low shear interval (0.1 s^–1^) for 60 s, a higher shear (30 s^–1^) interval closer to 3D print processing rates for 60 s, and a low shear interval (0.1 s^–1^) for 600 to determine the time for and extent of viscosity recovery. Methods for the modulus recovery experiments^50^ involved testing at 10 rad/s for all 3 intervals; the first interval applied strain (0.1%) within the LVR for 60 s, the second interval applied strain (10%) outside the LVR for 60 s, and the third interval reapplied the strain (0.1%) within the LVR for 600 s. The initial interval provided an indication of baseline viscosity (or modulus), the second simulated higher shear during extrusion, and the third interval provided a recovery period for longer times since deposition on the printer build platform occurs for a longer period than extrusion.

### 2.5 Aqueous Experiments

#### 2.5.1 Composite Processing, CAD Design, and 3D Printing

The test geometry was designed as a 35 mm diameter cylinder with a 4 mm height drawn in TinkerCAD and exported as an STL file. The file was sliced using Ultimaker Cura v4.11.0 (Figure S3) with a side wall thickness of 0.8 mm, consisting of 2 outer shell lines (0.4 mm nozzle). The layer height was 0.2 mm, and the print speed was 40 mm/s. The infill was 20% gyroid with the top and bottom shells removed to provide a high-surface-area object for photocatalysis studies. The sliced models were 3D-printed using an Ender3Pro (Creality, Shenzhen, China) equipped with a direct drive (SKU M3201, Micro Swiss, Ramsey, MN) to better extrude stiff and brittle composite filaments. The extrusion and bed temperatures were set to 200 and 70 °C, respectively. Two to three skirts were applied to the build plate prior to model deposition to overcome the low shear viscosity of the PLA–TiO_2_ composites^15^ and acquire consistent flow prior to model deposition.

#### 2.5.2 Photocatalysis Kinetics

The photocatalytic reference standard methylene Blue (MB)^51^ was used to determine the degradation rates and thus photocatalytic performance of printed structures from two of the more agglomerated (TRMT#1, TRMT#5) and less agglomerated (TRMT#4, TRMT#6) filaments. Triplicated prints (*n* = 3) from each treatment were placed into 40 mL glass crystallization dishes containing 40 mL of buffered (alkalinity = 180 mg/L as CaCO_3_) reconstituted freshwater, formulated as a high hardness surface water surrogate in accordance with U.S. Environmental Protection Agency methods.^52^ The water was spiked at an initial concentration (*C*_0_) of 1 mg/L MB and allowed to equilibrate overnight (dark; 4 °C). Each experimental unit was filled to 40 mL and placed on an orbital table (VersaOrb2, CHEMcell, Chemical Glass Life Sciences, Cat#CLS-4021–100, Vineland, NJ) set to 40 rpm under a simulated broad spectrum natural light source (ReptiSun 5.0; Zoo Med Laboratories, Inc.; San Luis Obispo, CA): 65% visible light (λ ∼ 400 to 750 nm); 30% UVA (λ ∼ 320 to 400 nm); 5% UVB (λ ∼ 280 to 320 nm). The intensity of this light source was well characterized at the same distance from the air–water interface (20–21 mm),^15^ and light exposure was calculated and summarized by total hours exposed and cumulative light intensity (MJ/m^2^). All testing was in an environmental room (Darwin Chambers, St. Louis, MO) set to 21 ± 2 °C and 80 ± 5% humidity. Degradation of MB was quantified using a standard curve using a photospectrometer (Thermo Scientific, Genesys 5) at λ = 664 nm. Degradation rates were determined using the well-validated pseudo-first-order kinetic model used for MB,^53^ with the rates determined from the slope (*k*) as a function of time (*t*) using eq 5 and half-lives (*t*_1/2_) determined as ln(2)/*k*. The concentrations of MB initially spiked into the water (*c*_0_) and at any given time point (*c*_*i*_) were entered into eq 5.

5 $$k=\frac{-\ln\left(c_{i}/c_{0}\right)}{t}$$

## 3 Results and Discussion

### 3.1 Composite Characterization

The agglomerate size range of the TiO_2_ (19–25 nm primary particle size) in ultrapure water prior to compounding was 1125–35,000 nm without sonication and 113–75,000 nm with sonication (Figure S2). It is well known that TiO_2_ heavily agglomerates in circum-neutral surface waters with environmentally relevant ionic strength,^47^ resulting in metastable agglomerates with larger effective diameters that consist of ≥70 nm primary aggregates^47,48^ and >300 nm agglomerates in polymer composites,^11,54^ which explain these larger size ranges.

SEC determined that the molecular weight of the acquired PLA pellets (*M*_n_ = 81.8 kDa, *M*_w_ = 124.5 kDa, *Đ* = 1.52) marginally decreased (while dispersity increased) after 1× extrusion through the twin screw (*M*_n_ = 80.6 kDa, *M*_w_ = 130.0 kDa, *Đ* = 1.61) and further decreased with 2× (*M*_n_ = 78.5 kDa, *M*_w_ = 127.8 kDa, *Đ* = 1.63) and 3× (*M*_n_ = 72.8 kDa, *M*_w_ = 123.9 kDa, *Đ* = 1.70) extrusions (Figure S4). The dRI data have an elution time shift to higher values, suggesting a decrease in molecular weight due to polymer chain scission and/or degradation from repeated thermal and shear exposure during processing. PLA degradation is relatively sensitive to repeated processing,^55^ with an associated decrease in viscosity^42^ and can further degrade in association with TiO_2_.^56^

### 3.2 Thermogravimetric Analysis

The percent mass loading of TiO_2_ in the composites was determined after increasing the temperature (10 °C/min to 600 °C) until all PLA was removed and only TiO_2_ mass remained (Figure S5). The eight TiO_2_ composite treatments (nominally 20% w/w) had similar measured loadings, ranging from 18 to 24% w/w (Table 1); this is important for comparability in rheological and photocatalytic performance. Additional extruded filaments using TRMT#5 and TRMT#6 twin screw process parameters (Table 1) prepared with higher TiO_2_ loadings (measured at 27% w/w) were used for a subsequent comparison of the relative impacts of TiO_2_ loading on viscosity.

### 3.3 Assessment of Agglomeration and Dispersion

#### 3.3.1 TiO_2_ Distribution in the Polymer

Large TiO_2_ agglomerates (>20 μm diameter) in the filaments were assessed as the relative percentage of the total cross-sectional area of the filament and as the total number of agglomerates in the cross sections per mm^2^ (*n* = 5). The distribution and size of TiO_2_ in the filament cross sections indicated the presence of notably different agglomeration states between treatments (Figures 1 and S6) despite similar TiO_2_ mass loadings (Table 1). Both the total area of the filament composed of TiO_2_ agglomerates (Figure 1A) and the total number of TiO_2_ agglomerates/mm^2^ (Figure 1B) indicated 2× extrusion reduced agglomeration. Note that the relative differences in bar magnitudes between 1× and 2× filaments in Figure 1 were smaller for the number of agglomerates (Figure 1B) compared to the total agglomerate area (Figure 1A). This indicates that 2× extrusion was more effective at reducing the size of large agglomerates than at reducing the number of agglomerates.

**Figure 1:**
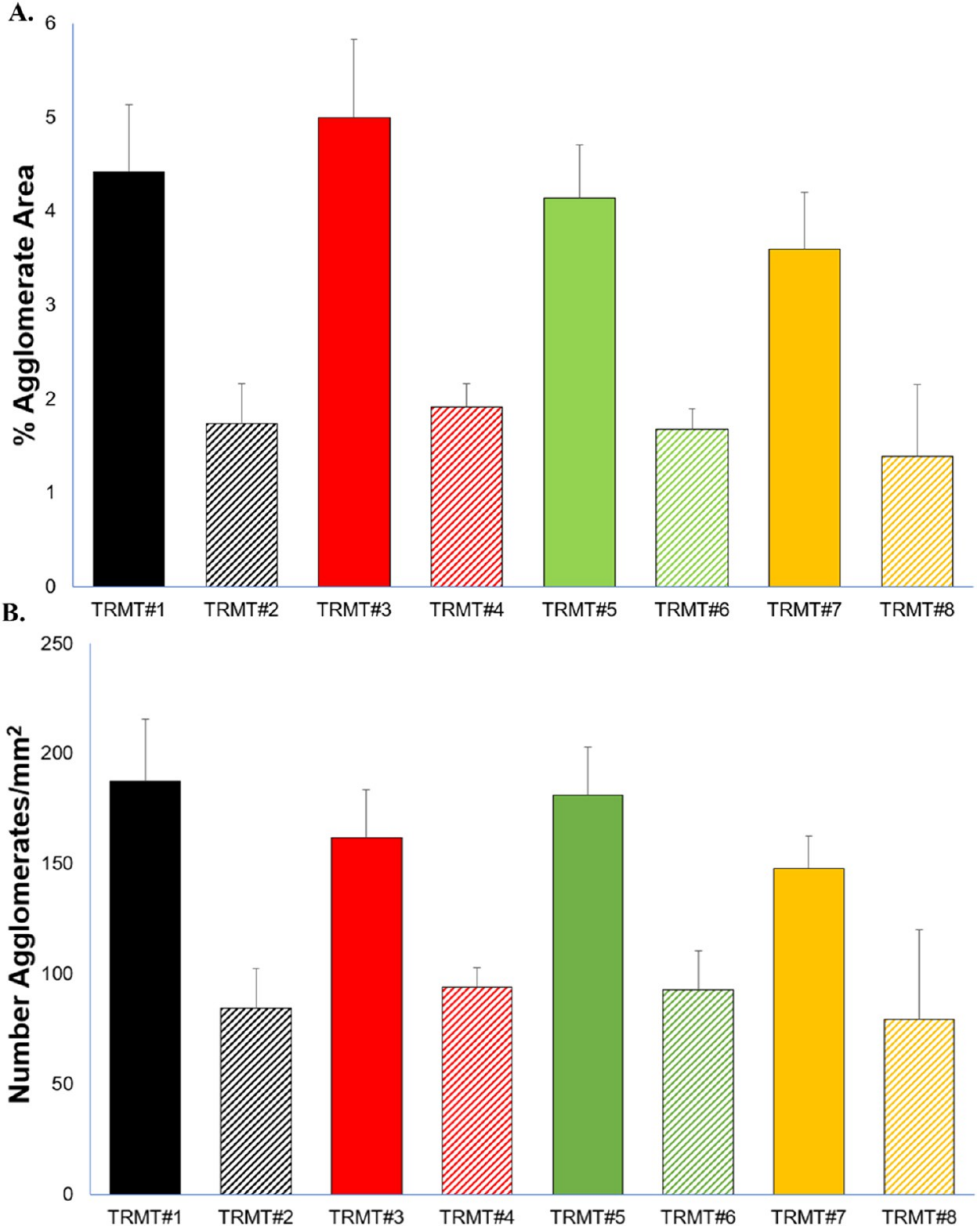
Percent area of the (A) filament cross sections that consisted of TiO_**2**_ agglomerates and (B) total number of agglomerates. Treatment 1 (TRMT#1) = 180 °C screw temperature, 300 rpm screw speed and 1× extrusions (180C-300 rpm-1×); TRMT2 = 180C-300 rpm-2×; TRMT3 = 220C-500 rpm-1×; TRMT4 = 220C-500 rpm-2×; TRMT5= 180C-500 rpm-1×; TRMT6 = 180C-500 rpm-2×; TRMT7 = 220C-300 rpm-1×; TRMT8 = 220C-300 rpm-2× (see Table 1). Solid bars represent 1× extrusion and hashed bars represent 2× extrusions.

Statistical analysis indicated that the number of times extruded was the most important factor determining both total agglomerate area and number of agglomerates. While there was significant interaction between temperature and screw speed impacting the total agglomerate area, this interaction did not significantly affect the total number of agglomerates per cross-sectional area. Generally, at higher temperatures and screw speeds, the residence time and viscosity of the polymer composite in the barrel were both too low for sufficient TiO_2_ deagglomeration and mixing. At lower temperatures, screw speed was unimportant, providing sufficient shear at higher polymer viscosity to mix the agglomerates. A detailed description of the statistical results (F-statistics and p-values) is provided in the Supporting Information. The interaction between screw speed and temperature was likely important only for reducing agglomerate area (and not number of agglomerates), since the shear forces applied to this system were sufficient to break apart large agglomerates but insufficient to further reduce agglomerate size once they were reduced to a critical size. Two extrusions coupled with higher temperature (220 °C) and slower screw speed (300 rpm) reduced the total agglomeration and total number of agglomerates. While the number of extrusion cycles was the most dominant factor, previous research reported that within a single processing cycle, high screw rotation (shear rates), higher temperature profile, and longer residence time are most impactful for improving dispersion of nanocomposites.^27,28^

#### 3.3.2 Treatment Impacts on Thermal Properties and Crystallization

The melting and crystallization behavior of the processed PLA–TiO_2_ composites were compared to that of neat PLA, since they may impact the printability of composite filaments.^57,58^ The first and second DSC heating curves (Figure 2) and the associated parameters (Table S1) are provided. TiO_2_ agglomeration did not impact the *T*_g_ (60–65 °C) in the predominantly amorphous PLA here or previously.^12,38,59^ The composites exhibited an exothermic transition (115–135 °C) associated with PLA cold crystallization, as commonly observed during slow crystallization of semicrystalline polymers.^54,59^ TiO_2_ altered the cold crystallization temperature (*T*_cc_) and enthalpy of cold crystallization (Δ*H*_cc_) of PLA (Table S1). The amorphous PLA chains likely had insufficient time to reorient in 1× extrusions at 180 °C and 300 rpm (TRMT#1), resulting in a lower intensity of the cold crystallization exotherms. The reduced PLA chain mobility can be attributed to both longer chain lengths (1× had higher *M*_w_; Figure S4) and the TiO_2_ filler, causing physical hindrance.^60^ Also, TiO_2_ surfaces have hydroxyl groups that can associate with PLA carbonyl groups,^61^ resulting in reduced chain mobility and crystallization kinetics.^62^

**Figure 2:**
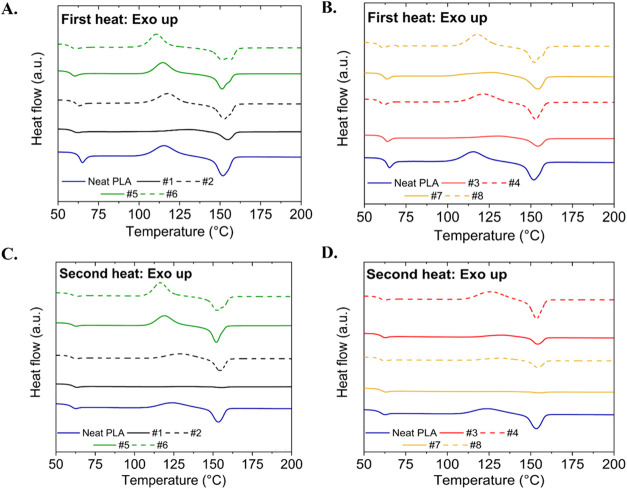
Differential scanning calorimetry melting endotherms obtained from a heat–cool–heat nonisothermal experiment for the PLA–TiO_**2**_ composite filaments prepared at (A) 180 °C (first heat), (B) 220 °C (first heat), (C) 180 °C (second heat), and (D) 220 °C (second heat). The curves were shifted vertically to allow visual comparison.

Increasing the screw extrusion temperature, speed, and/or reprocessing cycles (1× to 2×) resulted in reappearance of the cold crystallization exotherm, likely due to chain scission during multiple reprocessing cycles from exposure to higher temperatures and shear rates. This is supported by the reduction in *M*_w_ for 2× extruded PLA composites (Figure S4) and TiO_2_ nucleation effects.^63^ Nucleation was enhanced when the composites were 2× extruded (reduced % agglomerate area relative to 1×; Figure 1) and processed at higher temperatures or shear rates (screw rpms). The samples processed at 220 °C have higher Δ*H*_cc_ compared to their 180 °C counterparts, which suggests greater chain scission at higher temperatures that allows greater PLA mobility of shorter chains that more readily arrange into crystallizable fractions. For the 2× samples, the increased dispersity of the polymer compared to neat PLA can also be an important factor in determining the crystallizability and kinetics in the samples (Figure S4). Thermal history has a strong influence on the crystallization behavior of PLA,^55^ as shown in differences observed between first (mimicking the solidification stage of filament following twin screw extrusion) and second DSC heats (Figure 2). Additional observations following cold crystallization are provided in the Supporting Information.

Figure 3A highlights the impact of processing conditions on the total crystalline content of the PLA–TiO_2_ composites; the degree of crystallinity between the first and second heats was significantly correlated (*r* = 0.736, *p* = 0.037) and grouped according to 1× or 2× extrusions. The degree of crystallinity of PLA increased with addition of TiO_2_; this was observed for both the directly extruded filaments (1st heat), where neat PLA was 6.1% compared to the range in PLA–TiO_2_ filaments (4.9–15.9%) and when thermal history was removed (2nd heat), where neat PLA was 2.4% compared to the 2× extruded PLA–TiO_2_ composites (5.5–16.7%) and the 1× extruded PLA–TiO_2_ composites (2.1–11.1%) (Table S1). At a constant processing temperature and shear rate, multiple extrusion runs resulted in an increase in crystallinity (i.e., shorter chains lead to greater crystallinity). In fact, the first degree of crystallinity was significantly negatively correlated (*r* = −0.850, *p* = 0.007) with total agglomeration (Figure 3B). This result along with the greater *T*_cc_ compared to neat PLA suggests that TiO_2_ particles act as nucleating agents.^12,38^ The higher degree of crystallinity indicates that the fillers did not impact the heterogeneous nucleation (slower process due to chain aggregation) of the PLA chains.^64^ Previous work indicates that while lower loadings (e.g., <5%) of TiO_2_ can enhance crystallization by nucleation, higher amounts of TiO_2_ may overpack the system and restrict chain mobility, reducing crystallization potential.^54,59^ This expected crystallization did not appreciably impact printability but remained informative for understanding processing impacts (e.g., rheological recovery) and direct or indirect correlations to states of TiO_2_ agglomeration (Figure 3).

**Figure 3:**
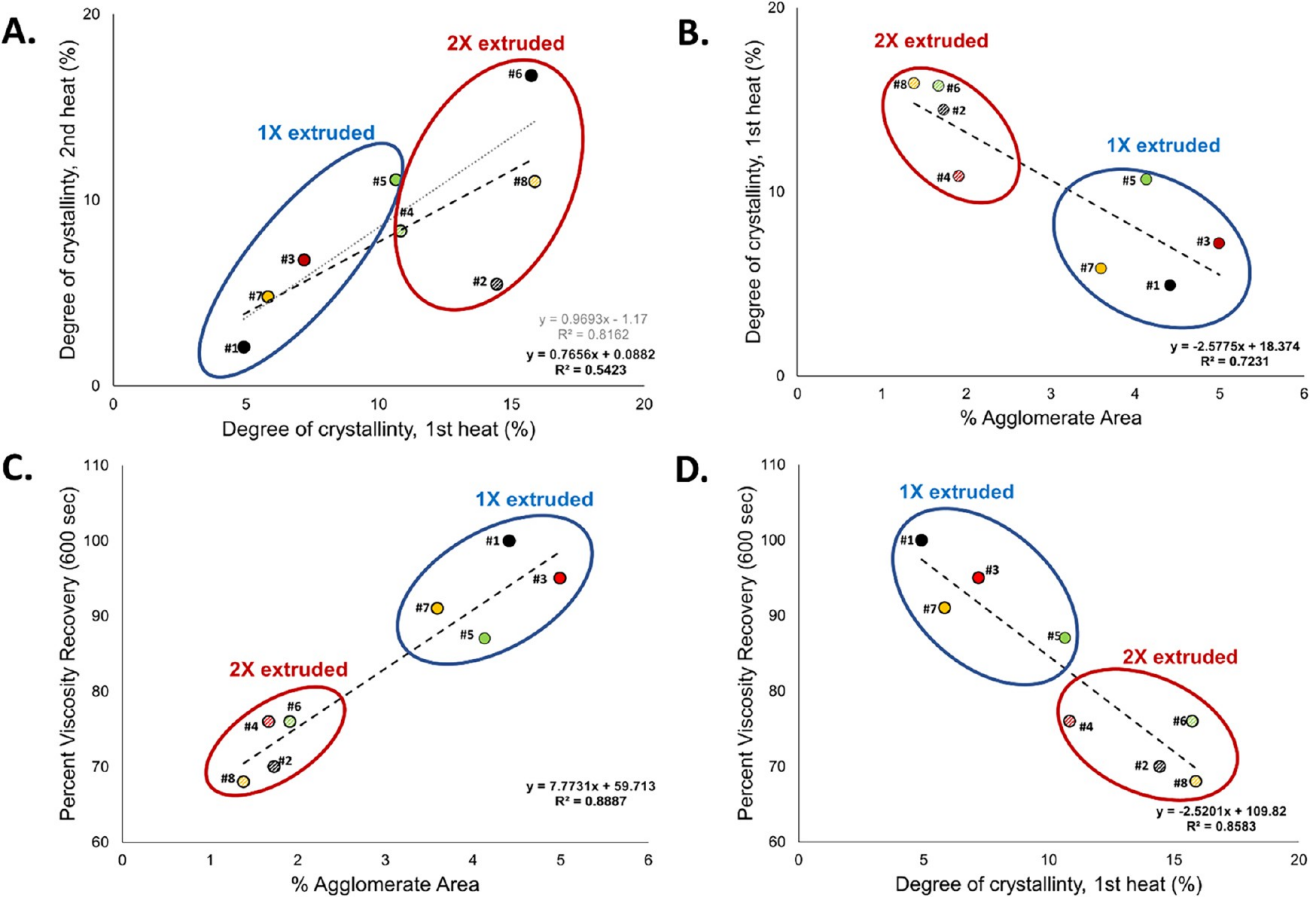
Correlations between (A) degree of crystallinity (1st and 2nd heats), (B) degree of crystallinity (1st heat) and percent agglomerate area, (C) percent viscosity recovery and degree of crystallinity (1st heat), and (D) percent viscosity recovery and percent agglomerate area for TiO_**2**_ loadings ranging from 18 to 24% w/w. Solid points and blue circles indicate 1× extrusions, while hashed bars and red circles indicate 2× extrusions.

#### 3.3.3 Treatment Impacts on Rheology

In 3D printing, a rapid transition from flow behavior (*G*″ > *G*′) at higher rates to allow successful extrusion to more solid-like behavior (*G*′ > *G*″) at lower rates during postextrusion deposition onto the build plate is desirable for rapid structural solidification and dimensional accuracy.^57,65^ This is particularly important for MatEx-Direct Ink Write extrudates, since the process is isothermal and depends on rapid, low-rate-induced fluid–solid transition for build solidification.^66^ MatEx-FFF is nonisothermal, and solidification is not solely reliant on the higher viscosity achieved through the reduced processing rates that occur post extrusion; FFF also benefits from a higher viscosity obtained during postextrusion cooling.

To understand and overcome PLA–TiO_2_ print processing issues, including nozzle clogging we previously observed,^15^ a rheological comparison was conducted at an extreme range of processing temperatures. The complex viscosity of the PLA–TiO_2_ filament tested at four temperatures (Figure 4A) indicated less rate dependence at low frequency at the lowest temperature for which flow was poor (125 °C) but strong shear thinning across all tested frequencies at higher temperatures (170, 200, 220 °C); more importantly, testing at progressively higher temperatures illustrates a trend of increasing importance of TiO_2_ on lower rate polymer viscosity. While neat PLA viscosity decreases with increasing temperature,^15^ the PLA–TiO_2_ composite showed higher viscosity at low frequencies (0.1–1 rad/s) when tested at 220 °C relative to 170 and 200 °C. The composites also had greater stiffness (*G*′), which was not rate-dependent from 0.1 to 1 rad/s (Figure S8), suggesting particle-dominated behavior enabled by the lower PLA viscosity at higher temperatures. This implies that the lower PLA viscosity at low frequencies (and higher temperature) allowed greater TiO_2_ interactions, leading to greater *G*′ and viscosity at lower rates. At higher frequencies, however, the viscosity of the composite at 220 °C was strongly shear thinning and decreased rapidly due to network breakup, hydrodynamics, and potential particle-induced lubrication of chains.^12,42,54^ The lower viscosity observed can be enhanced by PLA degradation in the presence of TiO_2_.^12^ We observed PLA degradation when tested at 220 °C based on the change from clear to light tan coloration, but a higher *G*′ at low frequencies. Lower PLA viscosity at higher temperatures (potentially further promoted by degradation) allowing greater TiO_2_ particle interactions in the higher loaded 33% composites provides an explanation for why we previously observed frequent nozzle clogging at higher temperatures during initial 3DP extrusion and retractions.^15^ Due to these observations, subsequent rheology and 3DP processing was conducted at 200 °C (rather than higher temperatures) for more consistent composite flow behavior and to reduce PLA degradation.

**Figure 4:**
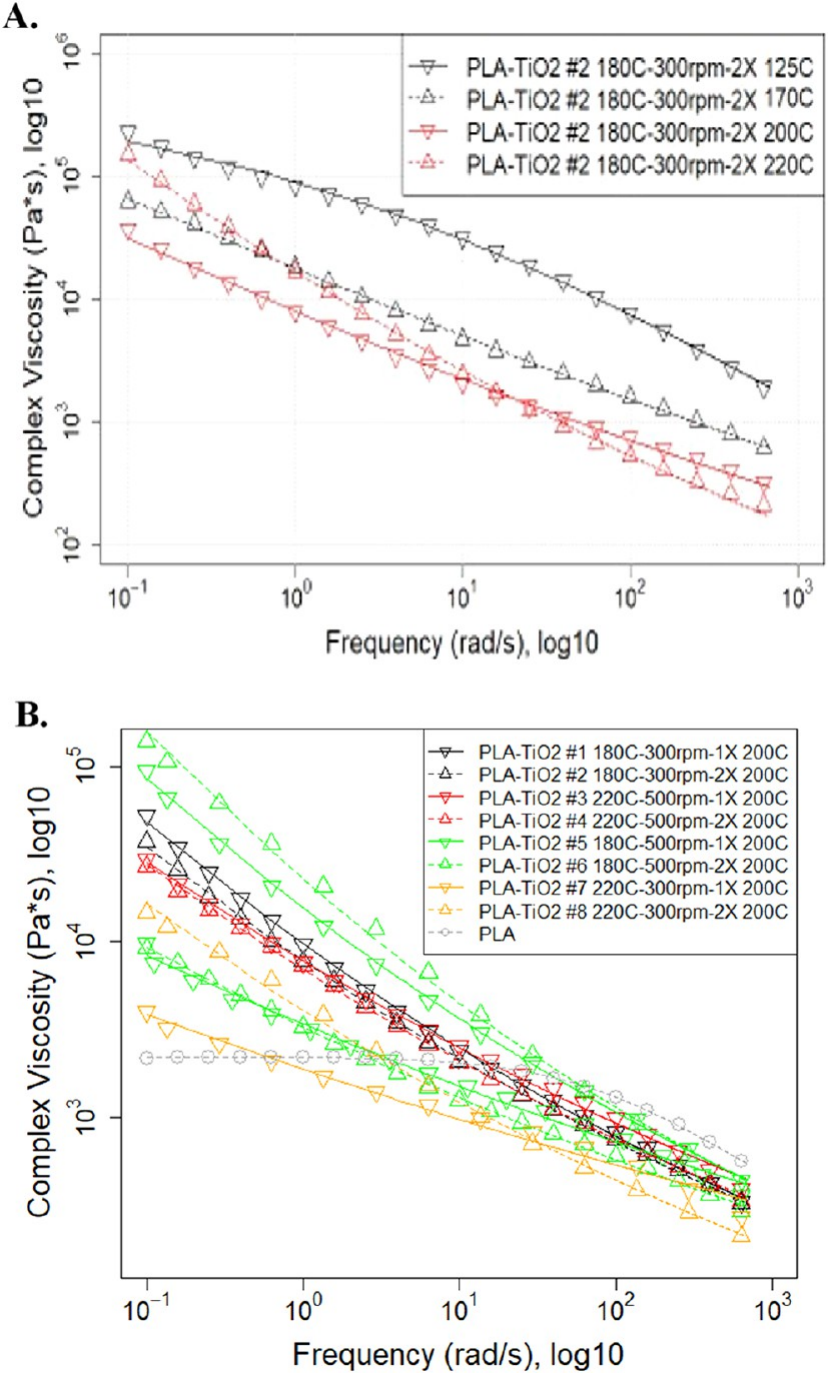
Small amplitude oscillatory shear experiments. Panel (A) shows a PLA filament with 24% TiO_2_ loading tested at different temperatures. Panel (B) shows the viscosity of the 8 different PLA–TiO_2_ filament treatments that ranged from 18 to 24% TiO_2_ (see Table S1 for measured values). A direct comparison of both filament treatments #5 and #6 (two different green plots) at lower (18–19%) and higher (27%) TiO_2_ loadings was tested to show the much higher viscosity caused by the greater loadings of TiO_2_ on the PLA filaments.

The viscosity of neat PLA at 200 °C had a Newtonian Plateau at low frequencies, followed by an area of shear thinning at higher frequencies (Figure 4B). The zero shear viscosity of PLA (2,240 Pa·s) was substantially lower than the low-frequency (0.1 rad/s) viscosity of all PLA–TiO_2_ composites (range: ≈3980 to 158,490 Pa·s). Greater TiO_2_ loading in PLA increased viscosity at low rates, as illustrated by the two different loadings (19 vs 27% w/w) tested for TRMT#5 and #6 (Figure 4B). Even comparing the similar loadings (18–24%), Kendal ranks of the low shear (0.1 rad/s) paired treatment viscosity averages (TRMT#1 and #2, TRMT#3 and #4, etc.) was ordered from high to low TiO_2_ loading (τ = 0.913, *p* = 0.071). Thus, the viscosity of treatment pairings (i.e., TRMT#1 and #2, #3 and #4, etc.) that were 1× versus 2× extrusions of the same material (equal TiO_2_ loadings) grouped together at low rates. Another observable trend was that when the viscosity of treatment pairings deviated (i.e., 27% TiO_2_ w/w TRMT#5 vs #6; TRMT#7 vs #8), the viscosity of the 2× extruded material was higher than that of the 1× extruded material. While, in context with the polymer, this may appear counterintuitive (i.e., multiple extrusions of PLA result in degradation, shorter chains, and less entanglement), it is explained by the composite behavior at these higher loadings shifting to control by the smaller-sized nanoparticle agglomerates and greater surface area for interactions and frictional forces.^67^ The trend of higher viscosity for the 2× extruded PLA–TiO_2_ can be explained by (1) better dispersed TiO_2_ facilitating more frictional particle–particle and particle–polymer interactions^42,46^ or (2) the higher degree of crystallinity seeded by the better dispersed TiO_2_ and promoted by shorter more crystallizable chains; a higher degree of crystallinity increases polymer viscosity even in the melt due to retained micromolecular structural differences from restricted volumes, albeit this is incrementally reduced with increasing processing temperature.^43,55^ Greater volume restriction associated with the degree of crystallization, rather than crystal size, is more important to observed increases in viscosity.^68^ At higher rates, the viscosity of all treatments, regardless of TiO_2_ loading or processing method, tended to converge. Modeled viscosity parameters are given in Table S2.

The 3ITT conducted further informs how the differences in agglomeration impact the viscosity and processing (including 3DP retractions) of the filaments. An almost immediate fluid–solid transition in polymer composites with high solid loading, as observed for 33% TiO_2_ in PLA (Figure 5A), is potentially problematic during FFF filament retractions due to partial pre-extrusion solidification in the liquefier at low rates, which increases the potential for nozzle clogging. Some thixotropy (Figure 5B,C) for FFF composite feedstocks may reduce the potential for nozzle clogging during filament retractions at the extrusion temperature in the liquefier, with additional reliance on cooling and associated increased viscosity in the stand-off region postextrusion for solidification of the build.

**Figure 5:**
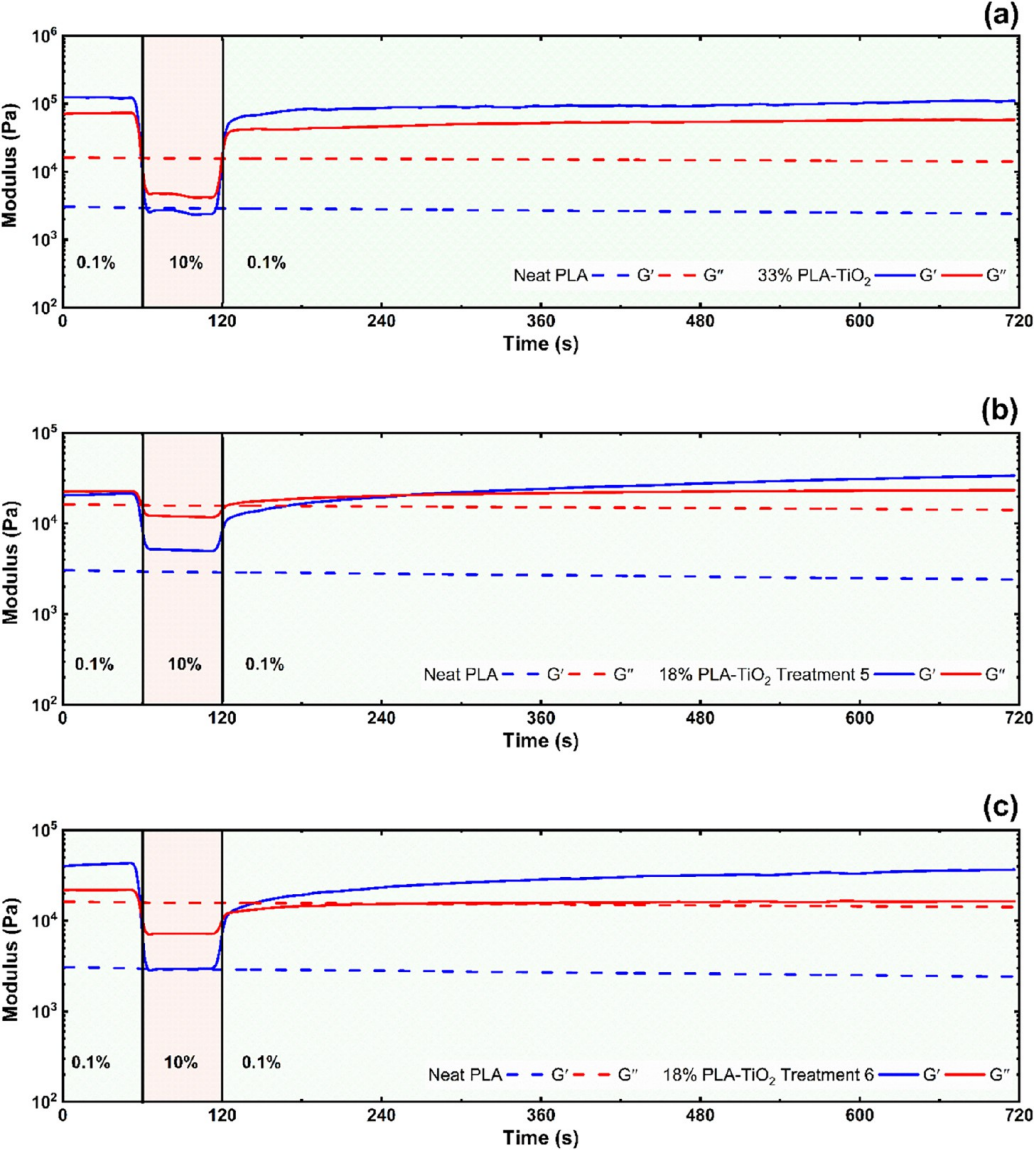
Modulus recovery for the composite filaments conducted at 200 °C in oscillation (10 rad/s). Data were from (a) reanalysis of PLA–TiO_2_ containing 33% TiO_**2**_ from Kennedy et al.,^15^ (b) Treatment #5 (1× extrusion), and (c) Treatment #6 (2× extrusion of Treatment #5). Treatments #5 and #6 contained approximately 18% TiO_2_. Interval 1 was low strain within LVR (0.1%), interval 2 was high strain outside LVR (10%), and interval 3 was low strain within LVR (0.1%) for modulus recovery. Dashed lines represent neat PLA behavior.

While nonisothermal conditions are dominant due to the design and time scales inherent to FFF processing, slow chain recovery after nozzle extrusion provides insights into the potential for hydrodynamically driven impacts on the structure, including TiO_2_ reagglomeration (or lack thereof). The 27% TiO_2_-loaded filaments vs 19% loaded filaments processed under TRMT#5 and #6 conditions are again shown for comparison (Figure S9). The reduction in viscosity from the first interval (low shear) to the second interval (higher shear) was related to (1) disentanglement and alignment of polymer chains, allowing them to slip past one another,^69^ and/or (2) breakup of agglomerate networks, allowing flow.^42^ The viscosity for most composites recovered to a plateau by 600 s, while the more agglomerated composite TRMT#1 recovered stochastically (Figure S9). Generally, the viscosity recovery clustered together according to 1× vs 2× extrusion was consistently greater for the 2× extruded filaments and was strongly correlated (*r* = 0.94, *p* < 0.001) with total percent agglomerate area (Figure 3C). Percent agglomeration explained 89% of the variability in percent viscosity recovery, and the remaining 11% was likely due to the different TiO_2_ loadings (ranging from 18 to 24%) and degree of crystallinity. The 2× extruded test materials likely had less recovery after high shearing due to the TiO_2_ being better dispersed (smaller, more dispersed agglomerates), which served to restrict chain mobility and consequently reestablishment of entanglements during the lower shear recovery period and due to the unrecoverable microstructural disruption of the higher degree of organization (crystallinity) within the 2× composites. The 1× extruded composites recovered more, as the large agglomerates had less impact on overall polymer matrix chain mobility (fewer particle–polymer interactions) and lesser crystalline organization. Recovery in viscosity was significantly negatively correlated with the degree of crystallinity (*r* = −0.926, *p* < 0.001) (Figure 3D), suggesting that the residual chain organization from the crystals played a role in increasing the low shear viscosity (and *G*′; see below) in the first 3ITT interval.

3ITT modulus recovery experiments showed that both the amount of TiO_2_ loading (Figure 5A) and the extent of TiO_2_ agglomeration (Figure 5B,C) impacted the relative magnitudes of *G*′ vs G″, determining flowability vs solidification during printing. This may dictate the size of the processing window for reliable printing and is relevant within the liquefier for extrusion and retractions that are required during the print processing of complex geometries.^15^ Generally, *G*′ was higher than *G*″ at lower strains (0.1%) for the 2× treatments (processed in the screw at 180 °C; Figures 5 and S10). At higher strains (10%), *G*′ decreased below *G*″, thus allowing these materials to flow and be extrudable for 3D printing but suggesting a smaller processing window relative to the 1× filaments. As noted previously, this result is due to particle-determined behavior (frictional forces) in the better dispersed composites that volume restrictions (even after melt) related to greater chain microstructural organization and crystallinity.^43,55^ With the caveat that solidification due to cooling in FFF occurs within seconds, a relatively higher modulus was observed for the 2× materials following deformation recovery after reduction from higher to lower rates, which, when occurring during postextrusion, aids rapid solidification of the printed object (see Figure 5A). This trend (*G*′ > *G*″ at lower rates) was less apparent for filaments processed in the twin screw at 220 °C (Figure S11), which have longer times for postextrusion polymer chain recovery. The higher the TiO_2_ concentration (up to 33% w/w^15^), the higher the modulus at low rates and the smaller the magnitude difference in *G*″ > *G*′ during higher rate extrusion (Figure 5A) that allows the material to be reliably extrudable and “printable”. The stiffness of this material (Figure 5A) causes it to recover its modulus almost immediately (*G*′ > *G*″), as compared to the more thixotropic material shown in Figure 5B, which likely results in the nozzle clogging discussed in Kennedy et al.^15^ These results further support limiting TiO_2_ loading to ≈20% for the present study to acquire more reliable printability (in addition to extrusion at 200 °C rather than higher temperatures where more particle–particle interactions occur).

### 3.4 Photocatalytic Performance

#### 3.4.1 Degradation Kinetics

Previous investigation^29^ determined low TiO_2_ loadings (≤2%) in PLA showed better anionic methylene orange degradation when produced by compression molding films relative to screw compounding and thermoforming. Other work provided evidence that higher loadings (from 2 to 40%) improve photocatalytic performance.^15,70,71^ To the authors’ knowledge, the current study is the first comparison of the photocatalytic efficacy of 3D-printed photocatalytic structures containing elevated TiO_2_ loadings (≈20% w/w) produced from different filament feedstocks that were prepared by altering twin screw extrusion process controls. TRMT#1, #4, #5, and #6 (Table 1) were selected for photocatalytic confirmation studies based on their higher (#1 and #5) and lower (#4 and #6) amounts of agglomeration (Figure 1). All four materials showed significant photocatalytic activity, reducing cationic MB 14 times faster than in the light treatment alone due to photolysis (neat PLA has no impact on MB concentrations^15^). Analysis of TRMT#5 (1×) and #6 (2×) allowed direct comparison of the number of extrusions of the sample material with the same TiO_2_ loading. TRMT#5 (1× extrusion) clearly had more TiO_2_ agglomeration than TRMT#6 (2× extrusion) (Figure 1), but the photocatalytic activity was similar between the two (Figure 6). The rates (0.37 ± 0.01, 0.32 ± 0.02, 0.34 ± 0.02, 0.35 ± 0.02 h^–1^ for TRMT #1, #4, #5, and #6, respectively) were generally within standard error and half-lives (1.9–2.2 h) for all tested materials (Table S3). Photocatalytic rates were slower than our previous study (0.79 h-1, *t*_1/2_ = 0.8 h) using lattices due to the difference in geometry and photocatalyst loading levels; while the depth in the water column was similar (20–21 mm), leading to similar light dissipation, the mass of the ≈20% w/w TiO_2_ gyroid disks was substantially lower (1.27 ± 0.19 g of the object; 0.25 g of TiO_2_) than the mass of the 33% w/w TiO_2_ lattices (5.27 ± 0.69 g object; 1.79 g TiO_2_) and consequently they contained less TiO_2_ (lower activity). This difference in geometry and photocatalyst loading was intended to slow degradation rates, thereby allowing for greater potential to observe differences among treatments.

**Figure 6:**
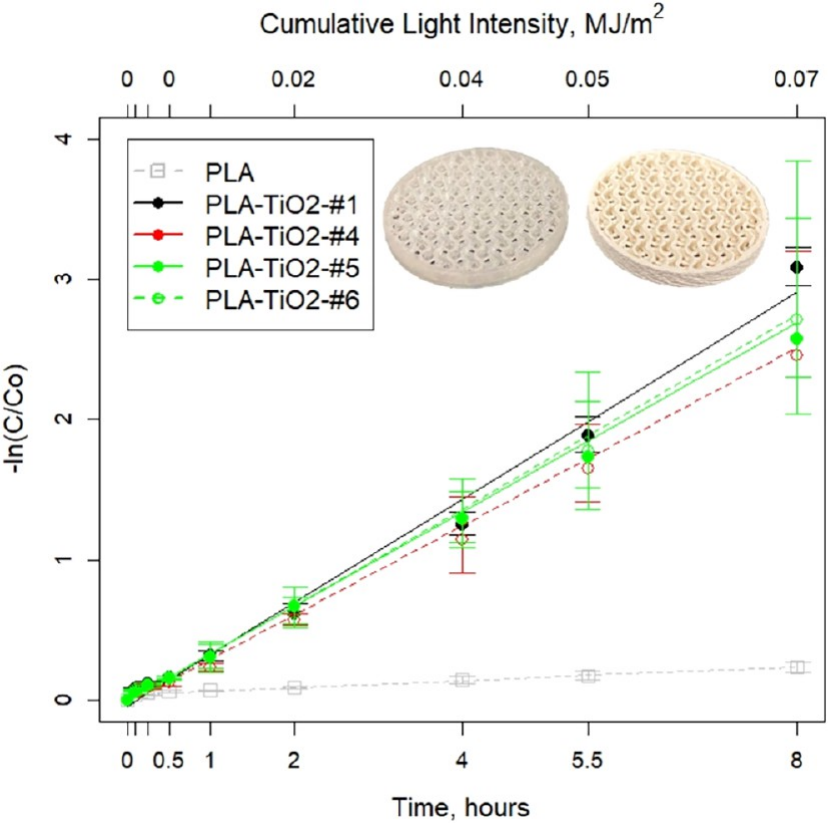
Photocatalytic degradation of methylene blue by PLA–TiO_**2**_ composites. The inset pictures show the neat PLA (left) and PLA–TiO_**2**_ composite (right) test structures.

#### 3.4.2 TiO_2_ Agglomerates in FFF Extrudates and Solidified Test Structures

Additional work was performed to provide insight into why different TiO_2_ agglomeration states did not appreciably impact photocatalytic rates. Cross-sectional SEM images of the extruded PLA–TiO_2_ filaments from the twin screw extruder (Figures 7 and S6), beads extruded from the 3D printer (Figure 8), and in the printed disks themselves (Figure S6) indicated that larger TiO_2_ agglomerates were present within the cross sections, but fewer were located along the walls (outer margins) of the extrudates. This observation was confirmed by XRM scans of three different cross sections of each of the four tested filaments after extrusion through the printer nozzle (Figure 7) and may be explained by geometric constraints (larger particles displaced by the presence of the extruder wall) and due to normal forces in non-Newtonian fluids. While Newtonian fluids lack normal forces and particles may migrate toward the wall, particles in confined, shear-thinning non-Newtonian fluids migrate away from the wall due to normal forces and a gradient of rate-dependent shearing.^72^ Axial forces and normal stresses measured in shear sweeps for neat PLA using a rotational rheometer confirmed nominal normal stress (and axial forces) in the Newtonian Plateau region of the viscosity profile, as expected, followed by dramatic increases once the PLA began shear thinning (Figure S12A). Larger particles in a constrained flow field travel away from the wall at a greater velocity than smaller particles in the shear-thinning polymer.^72^ The viscous forces while printing PLA at relevant shear rates (e.g., γ̇ > 30–400 s^–1^)^65^ are generally expected to predominate over inertial forces, since the Reynolds Number is low (≪1).^73^ The Weissenberg Number for processed PLA is greater than 1 and indicates elastic forces predominate over viscous forces due to the first normal force.^72^ Axial forces (and normal stresses) for neat PLA were higher than those for PLA–TiO_2_ (Figure S12B). This combination of geometric constraints and normal forces in the shear thinning processing region, supported by the literature and generated data, explains the reduced presence of larger agglomerates at the wall of the extruded roadways, resulting in the predominant presence of smaller TiO_2_ particles near the wall and presumably the observed comparable photocatalytic performance for all filament treatments.

**Figure 7:**
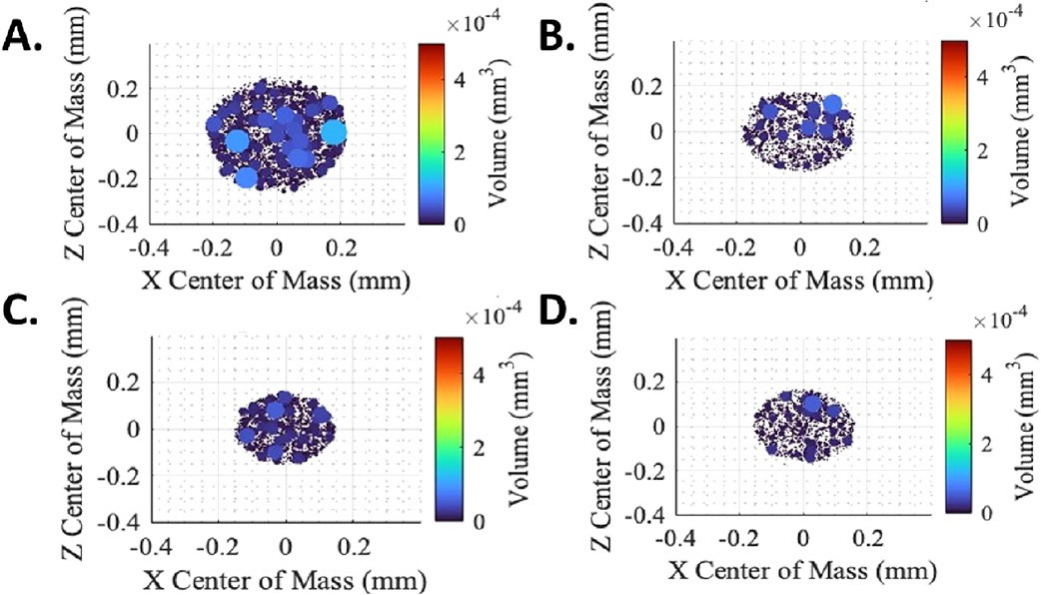
XRM scans of PLA–TiO_2_ 3D print extrudate cross sections from the (A) 1× extruded Treatment #1; (B) 2× extruded Treatment #4; (C) 1× extruded Treatment #5; and (D) 1× extruded Treatment #6. TiO_2_ agglomerates are color-coded by volume, with warmer colors indicating larger agglomerate volume and cooler colors indicating smaller agglomerate volume (see scale bars within the figure). Cross sections are dorsal-ventrally compressed due to deposition onto the build plate.

**Figure 8:**
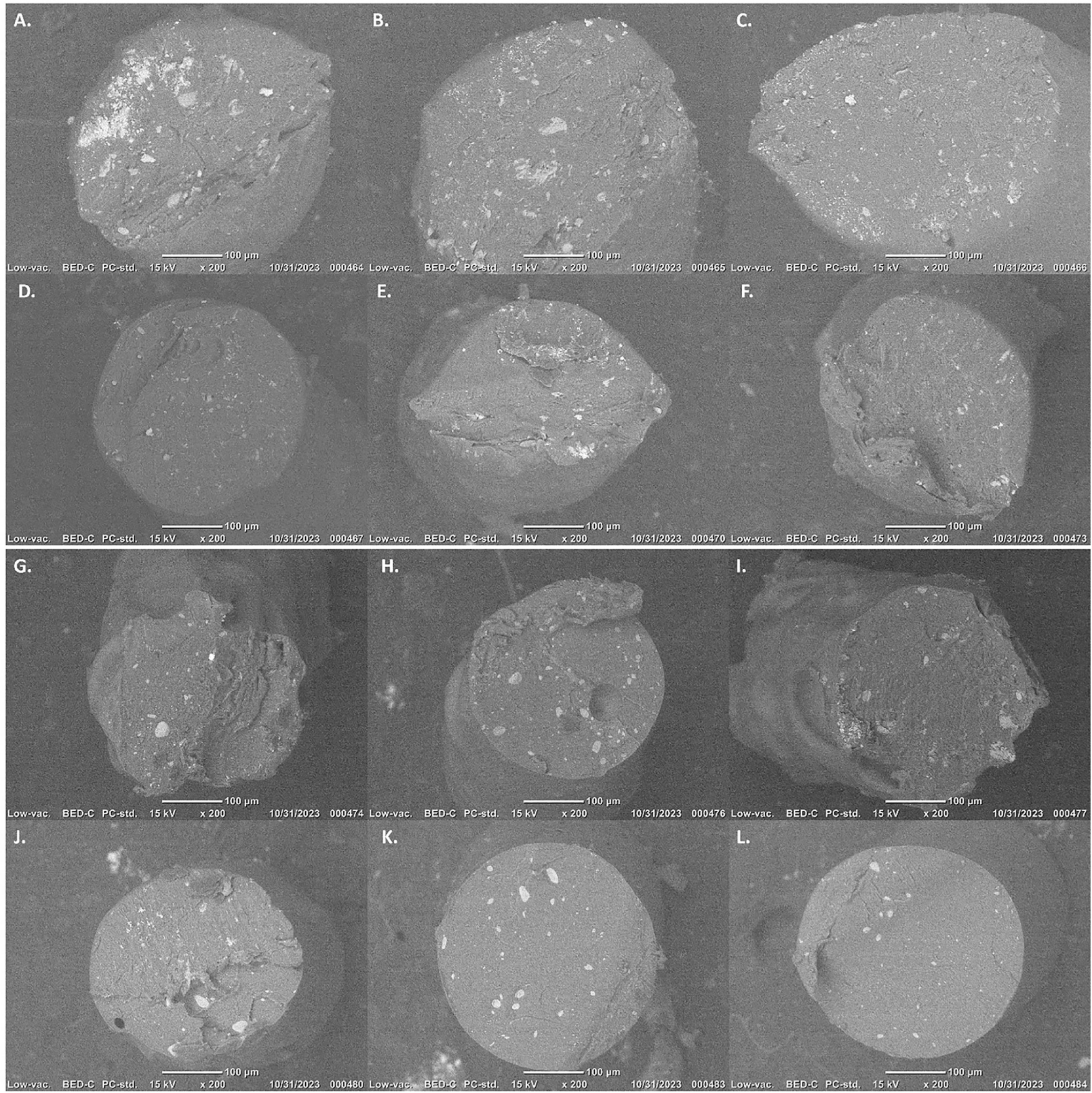
Scanning electron microscopy images of extrudates from the 3D printer collected before deposition. The panels summarize 1× extruded Treatment #1 (A–C), 2× extruded Treatment #4 (D–F), 1× extruded Treatment #5 (G–I), and 1× extruded Treatment #6 (J–L).

The clipped gyroid infill roadways (facing the light exposure) of the printed disks had fewer, readily visible large TiO_2_ agglomerates on the outer surfaces of the printed roadways relative to the filament and extrudate bead cross sections; there were discernible differences in the state of agglomerates between 1× and 2× extruded treatments (Figure S13). The printed disks from the 1× extruded filaments (TRMT#1:1.293 ± 0.032% total area, TRMT#5:1.258 ± 0.031% total area) had more apparent agglomerates than printed disks from the 2× extruded filaments (TRMT#4:0.197 ± 0.006% total area; TRMT#6:0.074 ± 0.019% total area). Again, the lower presence of TiO_2_ particle agglomerates on the surfaces of extrudates is consistent with research describing particle migration from the wall within constrained flow fields during extrusion of non-Newtonian polymer composites due to first normal forces.^42,72^ For example, a few large agglomerates are observable on the outer printed surfaces, but more agglomerates could be observed on the cut portion of prints (see Figure S13E,G).

Since there were no clear differences in MB degradation kinetics (Figure 6), we must conclude that the observed agglomeration in 1× printed samples was relatively unimportant in the overall photocatalytic efficacy of the various printed treatments. This result is likely due to adequate amounts of TiO_2_ within printed disks being sufficiently well dispersed by aggressive twin screw compounding, based on the homogeneous white coloration of all filament treatments, allowing sufficient surface for adsorption, radical formation, and MB degradation. Despite tendencies to agglomerate,^11,47,54^ the PLA C–O bonds promote interactions with TiO_2_ surfaces and the two have similar surface energies and thus lack strong interfacial tensions that would antagonize dispersion.^74,75^ Clearly, the more TiO_2_ is distributed outside of structures or coatings, the better the potential for photocatalytic performance.^11^ This observation suggests that the initial dispersion within the prepared filaments is important, as larger agglomerates may be prone to migrate from the side walls in the flow field during extrusion,^42^ making that material less available on the outer margins of the extrudate.

## 4 Conclusions

The three-tier study purpose was to determine how twin screw processing methods for embedding photocatalysts in 3D printable polymer impacted filler deagglomeration, thermal properties, processing, and consequently photocatalytic performance in aqueous media. Extensive characterization work was also performed to provide an explanation for why significantly reduced states of TiO_2_ agglomeration did not substantially improve photocatalytic performance.

The number of extrusions through the twin screw was the most dominant factor reducing total agglomeration and, to a lesser extent, the number of larger agglomerates in the extruded filaments. There was a significant interaction between screw speed and extrusion temperature, where at 220 °C (but not 180 °C), total agglomeration and number of agglomerates were reduced at 300 rpm based on the breakup of large agglomerates, but did not further reduce the number of agglomerates once they were reduced to a critical size. Overall, agglomeration was minimized by two extrusions at the higher temperature (220 °C) and lower screw speed (300 rpm) due to the lower viscosity of the PLA, allowing TiO_2_ migration and lower speed offering longer residence time in the barrel. Greater crystallinity was observed when the TiO_2_ dispersion was improved, likely due to seeding effects providing greater availability of smaller agglomerates and aggregates or more crystallizable PLA due to chain scission resulting from multiple processing cycles. The total loadings of TiO_2_ in PLA were more important for determining viscosity than the state of agglomeration of the tested filaments. Based on previous rheology data, we hypothesized that a 3D printing highly loaded (33% w/w) PLA–TiO_2_ filament at a higher temperature (215 °C) would improve extrusion and print consistency by reducing the viscosity of the composite in the nozzle, especially during lower rates, including filament retractions.^15^ However, the SAOS comparison of higher *G*′ at low rates, *G*′ > *G*″ relative magnitudes, and crossovers at low rates and 3ITT experiments provided evidence of greater particle-dominated behavior at 220 °C relative to 200 °C; this suggests more potential for printing inconsistencies at low rates (and higher temperatures) and increased potential for nozzle jamming. We also qualitatively observed browning (potentially thermo-oxidation and/or slight degradation) in PLA coloration (see Figure S14 for the image) when processing the PLA–TiO_2_ composites for more than several minutes at higher temperatures (later quantitatively determined to be minor in heat and hold TGA analysis; see Figure S14). Minor chain scission and degradation are supported in the literature at prolonged processing temperatures just above 200 °C^76^ and by our SEC data, showing a reduction in molecular weight with prolonged thermal processing (Figure S4). Therefore, 200 °C was better for reliable printing of the test structure for mass production, considering the larger amount of materials required for the environmental study scale-up.

While the state of agglomeration showed notable impacts on the thermal properties and degree of crystallinity, all ≈20% TiO_2_ materials printed relatively easily when print temperatures were reduced to 200 °C. Further, all tested filaments with varying TiO_2_ agglomerate states showed similar photocatalytic performance, which suggests that much of the TiO_2_ was sufficiently dispersed and distributed by twin screw extrusion and the printed structures to achieve acceptable photocatalytic properties. While image analysis of the printed structures used in photocatalysis testing showed that the filaments with greater agglomeration retained more agglomerates in the printed structures, substantially fewer agglomerates were observed on the surface of printed structures due to larger agglomerates migrating to the interior of the printed structures, while smaller, better dispersed particle agglomerates were retained on the outer surfaces where photoactivity is critical. This observation was related to flow fields induced during polymer composite extrusion. Based on the well-established MB photocatalytic standard, these findings indicate that twin screw extrusion and subsequent printing of reasonably dispersed photocatalysts will be adequate for treating other chemicals including conventional and emerging contaminants, such as previous work with algal toxin degradation,^15,24^ per- and polyfluorinated substances,^22,23^ and other organic contaminants of concern.^8,11,16,21^

## Supplementary Material



## Notes

Additional experimental methods and results on the thermal properties of test materials; molecular weights of test materials before and after processing; dispersion properties and concentrations of the nanoparticles in the polymer composites; and robust statistical summaries are included as descriptive text, tables, figures, and photographs (PDF)

## ∇

Carbon and Composites Group, Chemical Sciences Division, Oak Ridge National Laboratory, Oak Ridge, Tennessee 37830, United States
