# Supplementary material for: Breaking Bad: Deagglomerating TiO2 in 3D Printable Polymer Composites for Photocatalysis in Environmental Media
Source: ACS Appl Mater Interfaces. 2026 Feb 16;18(7):11430–44. doi: 10.1021/acsami.5c23498 (PMC12954662; doi:10.1021/acsami.5c23498)
Supplement: Supplementary file 1 [file am5c23498_si_001.pdf]

Supporting Information

Breaking Bad: Deagglomerating TiO_2 in 3D Printable Polymer Composites for Photocatalysis in Environmental Media

Alan J. Kennedy[a,b], Arit Das[b,c,#], Christopher Williams[b,d], Lucinda Slattery[e], Stephen Martin[b,d], Matthew Hull[f], Christopher Griggs[a], Michael J. Bortner[b,c]*

[a] US Army Engineer Research and Development Center, Environmental Laboratory, Building 3270, 3909 Halls Ferry Rd, Vicksburg, MS 39180, United States

[b] Virginia Polytechnic Institute and State University, Macromolecules Innovation Institute, ICTAS II, Suite 130, 1070 Life Science Circle, Blacksburg, VA, 24061, United States

[c] Virginia Polytechnic Institute and State University, Department of Chemical Engineering, 635 Prices Fork Road, Goodwin Hall, Suite 245, Blacksburg, Virginia 24061, United States

[d] Virginia Polytechnic Institute and State University, Department of Mechanical Engineering, 445 Goodwin Hall, 635 Prices Fork Road, Blacksburg, Virginia 24061, United States

[e] US Army Engineer Research and Development Center, Geotechnical and Structures Laboratory, Building 6000, 3909 Halls Ferry Rd, Vicksburg, MS 39180, United States Vicksburg, MS 39180, United States

[f] Virginia Polytechnic Institute and State University, Institute for Critical Technology and Applied Science, 325 Stanger Street, Suite 410, Blacksburg, VA 24061, United States

[#] Current address: Carbon and Composites Group, Chemical Sciences Division, Oak Ridge National Laboratory, Oak Ridge, TN, 37830 USA

* email: mbortner@vt.edu

1 THERMAL PROPERTIES AND CRYSTALLIZATION

Following cold crystallization, all filament treatments exhibited similar melting endotherms (150-155 °C). Presence of TiO_2 did not impact composite T_m relative to neat PLA. However, the enthalpy of melting (ΔH_m) of the composites is dependent on TiO_2 presence and processing treatment (Supporting Table S- 1). Compared to neat PLA, ΔH_m was lower in the composites due to defect formation in the macrostructure in presence of TiO_2 that interferes with the crystallization process. The interfacial bond between the filler and PLA matrix immobilizes the amorphous fraction of PLA, slowing diffusion kinetics for crystallization [1]. The remaining crystalline domains in the composites melted between 150-155°C. A few 2X extruded composites exhibited multiple melting peaks, likely due to polymorphic crystals or varying lamellar domains [2, 3]. The composites prepared at 180°C and 500 rpm (2X) have a melting endotherm characterized by a slightly lower temperature peak at 151 °C that indicates melting of mesostable crystals formed during cooling[4]. This is followed by a recrystallization exotherm that forms comparatively more stable crystals melting at higher temperature (156 °C). This behavior is attributed to the melting-recrystallization-perfection (MRCM) phenomenon during PLA heating causing imperfect or small crystals that reorganize and exhibit the multiple melting peaks[5].

1.1 TiO_2 distribution in polymer

For total agglomerate area (Supporting Figure S- 7A,B), the number of times extruded was the most important factor ($F = 200.14$; $p = 0.001$), while screw speed had secondary importance but was not statistically significant ($p = 0.121$) and extrusion temperature was unimportant ($p = 0.934$). Two extrusions (2X) significantly reduced the total agglomerate area within the filament. There was a significant interaction ($p = 0.005$) between temperature and screw speed that impacted total agglomerate area. At 220 °C, the faster screw speed (500 rpm) resulted in more agglomeration related to faster travel though the barrel (i.e., insufficient mixing residency time[6, 7]) at lower viscosity (i.e., at the higher shear rates and higher temperature). At 180 °C screw speed was unimportant, likely due to the relatively higher polymer viscosity at lower temperature (see Rheology Interpretation Section below) dictating the de-agglomeration regardless of screw speed; however, the higher viscosity at 180 °C did not allow sufficient

dispersion and distribution of TiO_2 agglomerates to minimize total agglomerate area in the cross section. Thus, the lowest total agglomeration was attained after two extrusions at 220 °C and 300 rpm.

For total number of agglomerates (Supporting Figure S- 7C,D), two extrusions ($F = 91.59$, $p = 0.002$) was the most important factor and extrusion temperature was second most important ($F = 3.33$, $p = 0.165$). Since screw speed by itself ($F = 0.79$, $p = 0.438$) and the interaction between screw speed and temperature ($F = 0.58$, $p = 0.501$) were not important for determining number of agglomerates, they were rolled into the residual error to focus on the important factors. This resulted in two extrusions significantly reducing the number of agglomerates ($F = 104.64$, $p < 0.001$) and lower temperature being important ($F = 3.81$) for reducing number of agglomerates, albeit not technically statistically significant ($p = 0.108$).

Supporting Table S1. The melting and crystallization parameters for the different PLA/TiO_2 composites obtained from the first and second heating cycles of a typical non-isothermal DSC experiment.

Sample	Filler content (%)	Glass transition temperature (°C)	Enthalpy of cold crystallization (J g^{-1})	Peak cold crystallization temperature (°C)	Enthalpy of melting (J g^{-1})	Peak melting temperat ure (°C)	Degree of crystallinity (%)
From the 1st heating cycle							
Neat PLA	0	64.9	22.59	115.7	28.29	151.7	6.13
180°C_300rpm_1X	22	62.5	6.56	130.6	10.13	154.8	4.92
180°C_300rpm_2X	24	63.4	8.81	118.0	19.02	152.7	14.45
220°C_500rpm_1X	20	64.0	14.74	130.6	20.1	154.2	7.2
220°C_500rpm_2X	19	61.8	9.00	121.7	17.17	153.0	10.84
180°C_500rpm_1X	17	60.6	7.24	115.0	15.47	151.2	10.66
180°C_500rpm_2X	19	60.9	10.13	111.0	22.00	151.2<br>156.4	15.75
220°C_300rpm_1X	19	63.7	14.00	127.2	18.40	154.2	5.84
220°C_300rpm_2X	19	61.5	7.13	118.0	19.10	152.0	15.89
From the 2nd heating cycle							
Neat PLA	0	62.4	15.46	124.8	17.65	153.3	2.35
180°C_300rpm_1X	22	63.4	7.93	133.7	9.45	155.8	2.09
180°C_300rpm_2X	24	62.8	10.13	129.4	14.00	154.5	5.47
220°C_500rpm_1X	20	63.2	8.00	131.0	13.04	155.2	6.77
220°C_500rpm_2X	19	62.4	6.36	132.5	12.64	154.2	8.33
180°C_500rpm_1X	17	62.8	7.55	119.2	16.11	152.1	11.08
180°C_500rpm_2X	19	63.4	8.33	116.5	20.92	152.7	16.70
220°C_300rpm_1X	19	63.0	12.04	133.0	15.64	154.2	4.78
220°C_300rpm_2X	19	62.4	7.41	126.3	15.68	153.6	10.98

Supporting Table S2. Modeled viscosity parameters for the Carreau (Neat PLA) and Power Law ($PLA-TiO_2$ composites) Models in small amplitude oscillatory shear. experiments.

	K	n	η_0 (Pa*s, 10^X)	λ	a
PLA	NA	0.908 ± 0.005 ($p < 0.001$)	3.347 ± 0.002 ($p < 0.001$)	0.011 ± 0.001 ($p < 0.001$)	0.977 ± 0.040 ($p < 0.001$)
#1	3.990 ± 0.005 ($p < 0.001$)	0.930 ± 0.000 ($p < 0.001$)	NA	NA	NA
#2	3.891 ± 0.004 ($p < 0.001$)	0.933 ± 0.000 ($p < 0.001$)	NA	NA	NA
#3	3.891 ± 0.007 ($p < 0.001$)	0.941 ± 0.001 ($p < 0.001$)	NA	NA	NA
#4	3.841 ± 0.004 ($p < 0.001$)	0.937 ± 0.000 ($p < 0.001$)	NA	NA	NA
#5 (19%)	3.532 ± 0.010 ($p < 0.001$)	0.954 ± 0.001 ($p < 0.001$)	NA	NA	NA
#5 (27%)	4.192 ± 0.007 ($p < 0.001$)	0.930 ± 0.001 ($p < 0.001$)	NA	NA	NA
#6 (19%)	3.516 ± 0.004 ($p < 0.001$)	0.947 ± 0.000 ($p < 0.001$)	NA	NA	NA
#6 (27%)	4.357 ± 0.014 ($p < 0.001$)	0.923 ± 0.001 ($p < 0.001$)	NA	NA	NA
#7	3.277 ± 0.014 ($p < 0.001$)	0.960 ± 0.001 ($p < 0.001$)	NA	NA	NA
#8	3.608 ± 0.011 ($p < 0.001$)	0.933 ± 0.001 ($p < 0.001$)	NA	NA	NA

Supporting Table S3. Photocatalytic reduction rates.

Material	Slope	St. error	p	$t_{1/2}$ (h)
PLA	0.025	0.002	< 0.001	27.86
#1	0.370	0.011	< 0.001	1.87
#4	0.318	0.018	< 0.001	2.18
#5	0.337	0.019	< 0.001	2.06
#6	0.346	0.018	< 0.001	2.00

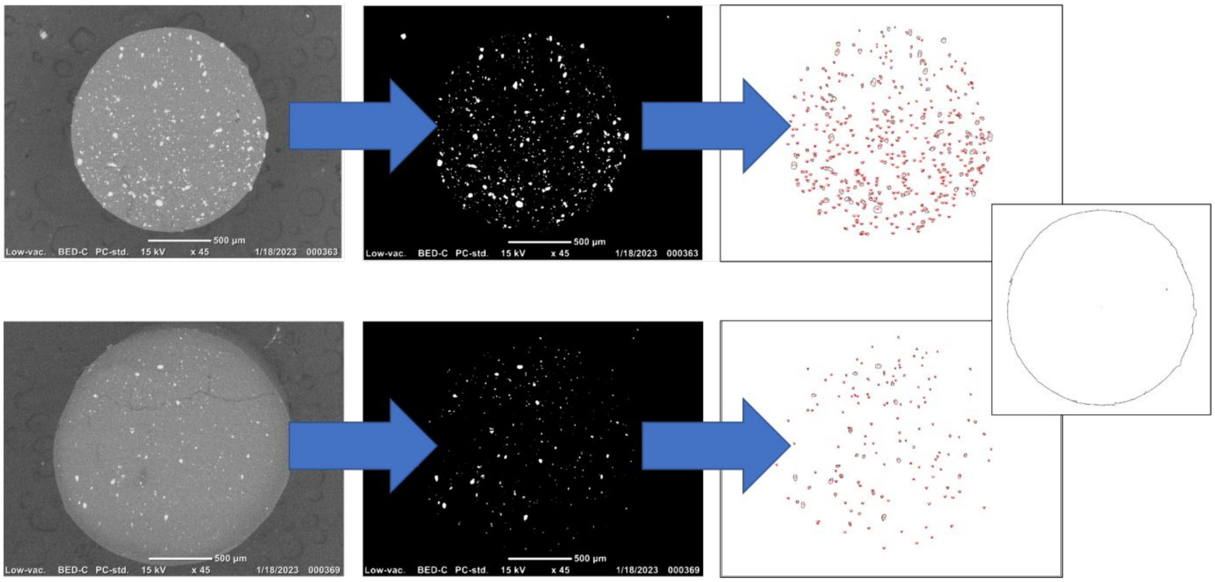

Supporting Figure S1. Process for imaging and analyzing cross sections of the PLA-TiO_2 filaments. The difference in agglomeration in 1X extruded Treatment #1 (top row of images) and 2X extruded Treatment #2 (bottom row of images) is shown. Each image was analyzed using ImageJ to determine extent of agglomeration. The first box on the left side of each row is the raw Scanning Electronic Microscopy (SEM) image where the TiO_2 agglomerates are shown in white. The next image shows the cleaned SEM image of only the TiO_2 agglomerates. The next image to the right shows identification of the area of each agglomerate. Finally, the smaller image on the far right indicates detection of the filament cross-section boundaries for percent area calculations.

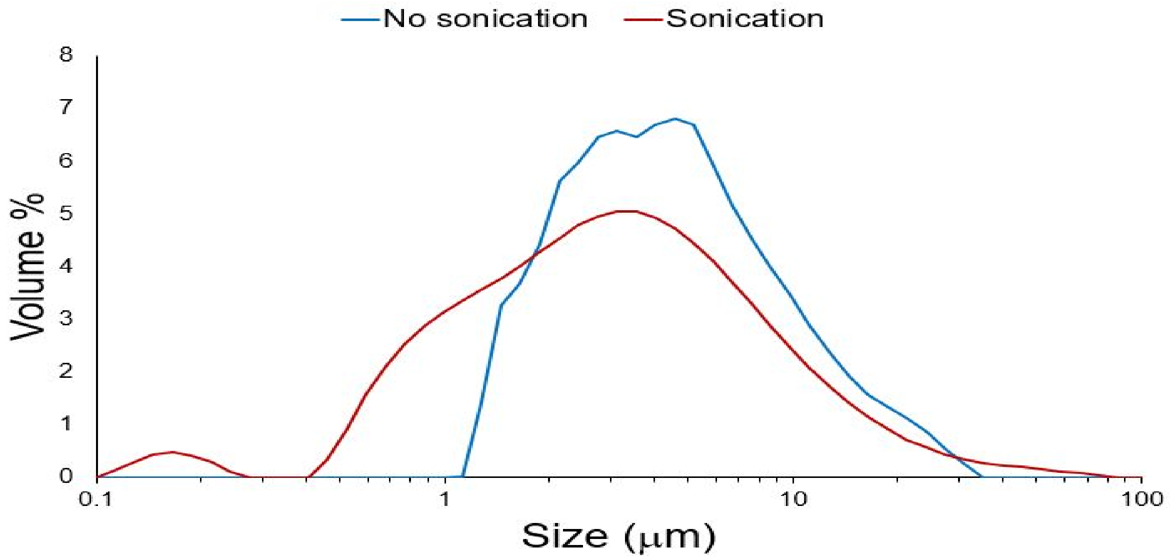

Supporting Figure S2. Size distribution of TiO2 with and without sonication.

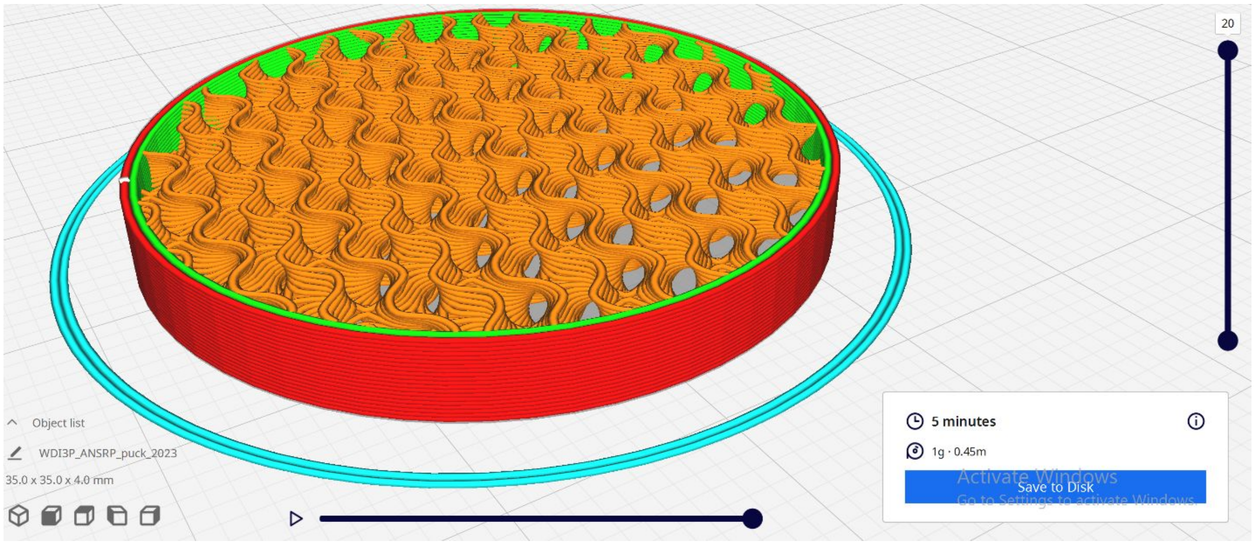

Supporting Figure S3. Sliced object for 3D printing providing high surface area for photocatalysis experiments. The test disk with 20% gyroid infill. The diameter and height were 35 and 4 mm, respectively. The layer height was set to 0.2 mm and therefore the model consisted of 20 layers.

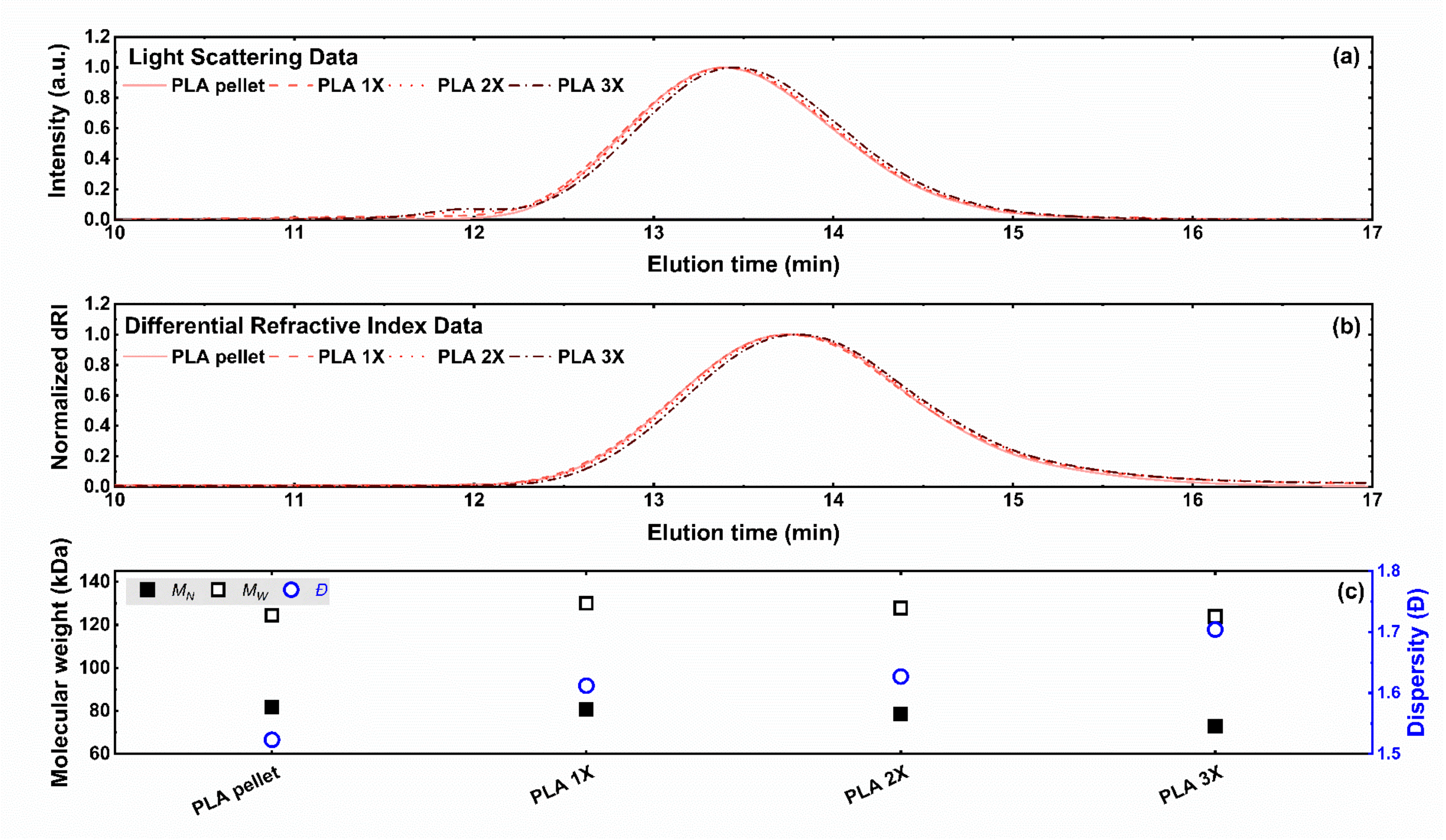

Supporting Figure S4. Size exclusion chromatography of PLA extruded multiple times summarized for the (a) light scattering detector, (b) refractive index detector and by (c) number average (M_n) and weight average (M_w) molecular weight and dispersity index (Đ).

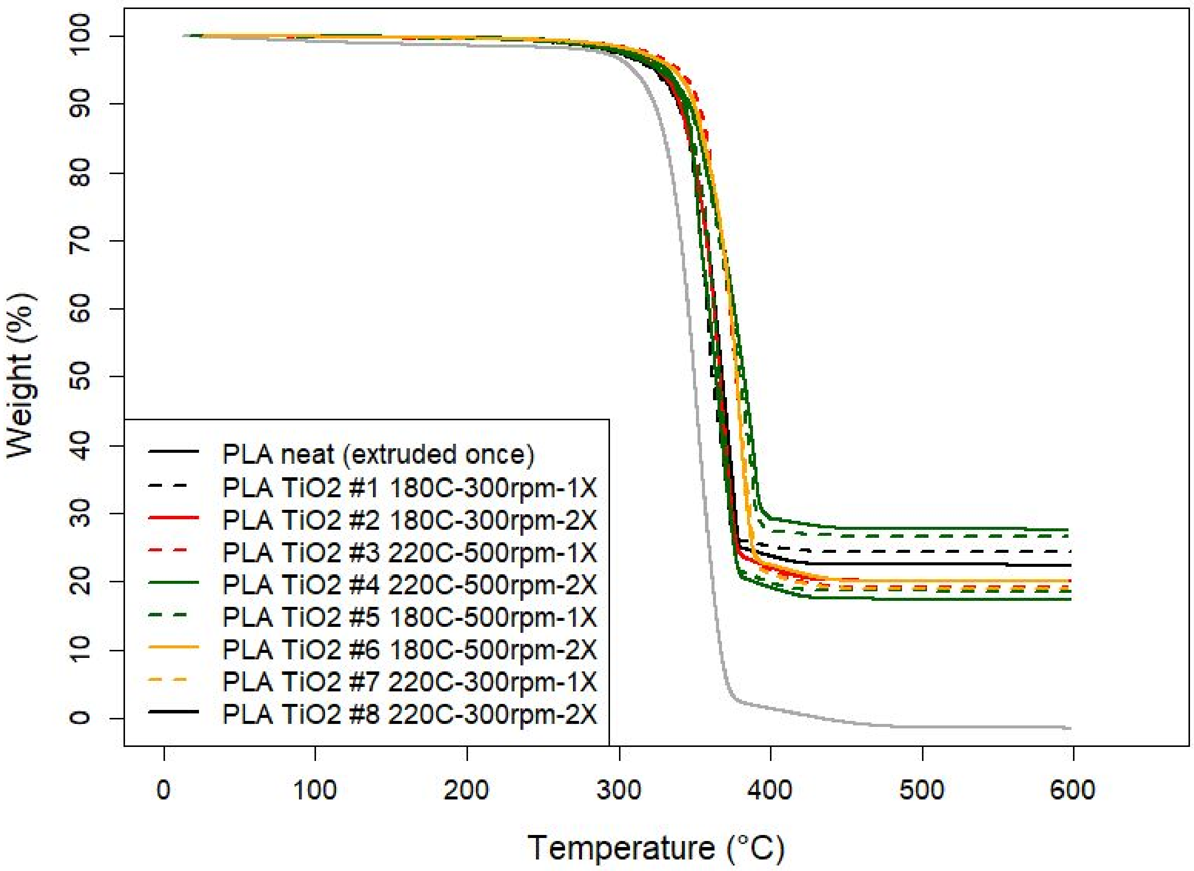

Supporting Figure S5. Thermogravimetric analysis of PLA and PLA-TiO2 filaments. Note that samples 5 and 6 appear twice to compare lower (≈20%) and higher (≈27%) concentrations.

A.

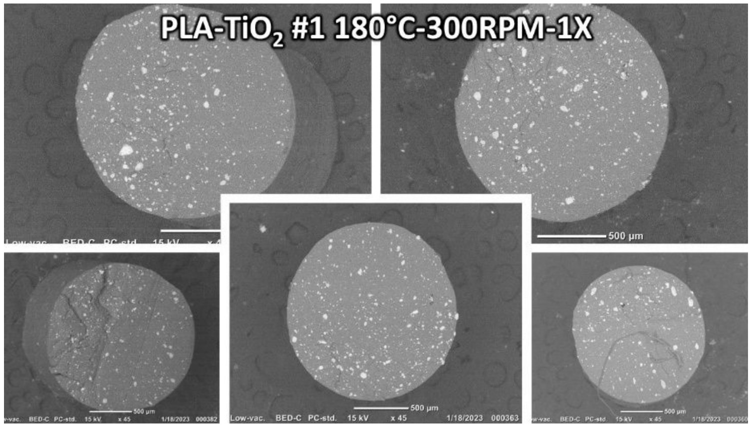
PLA-TiO2 #1 180°C-300RPM-1X

B.

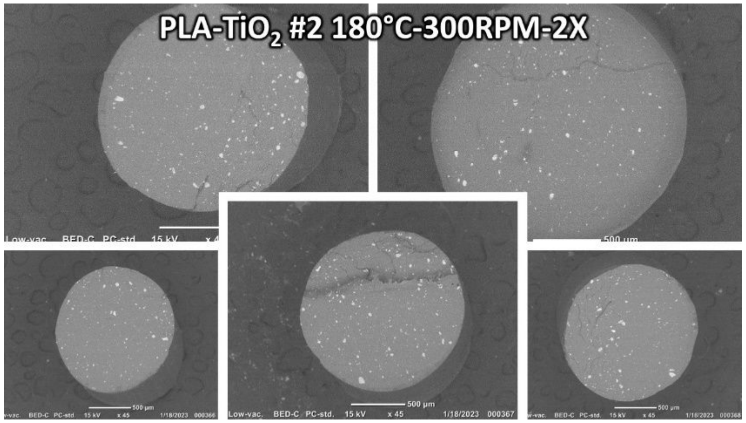
PLA-TiO2 #2 180°C-300RPM-2X

C.

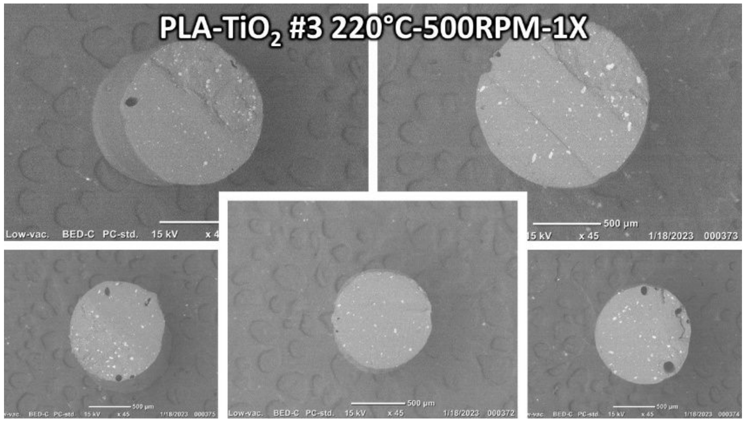
PLA-TiO2 #3 220°C-500RPM-1X

D.

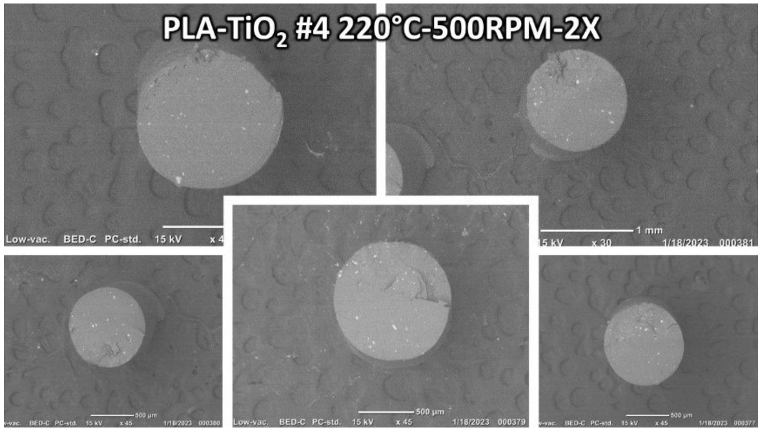
PLA-TiO2 #4 220°C-500RPM-2X

E.

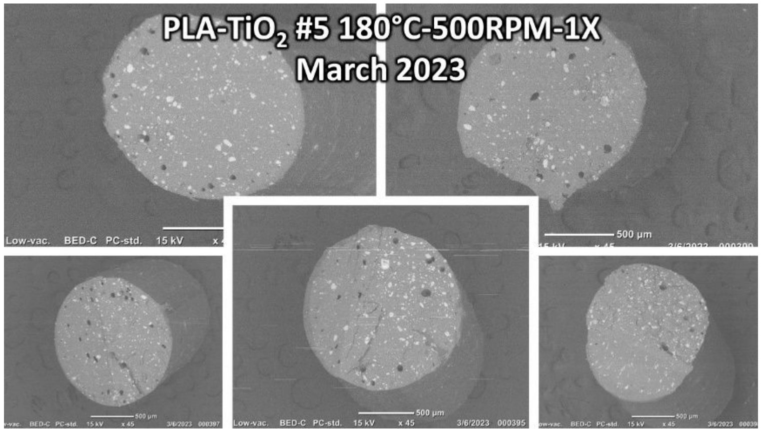

F.

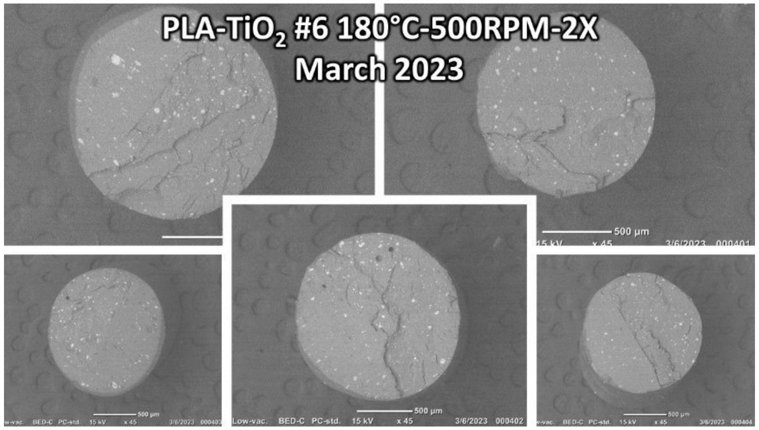

G.

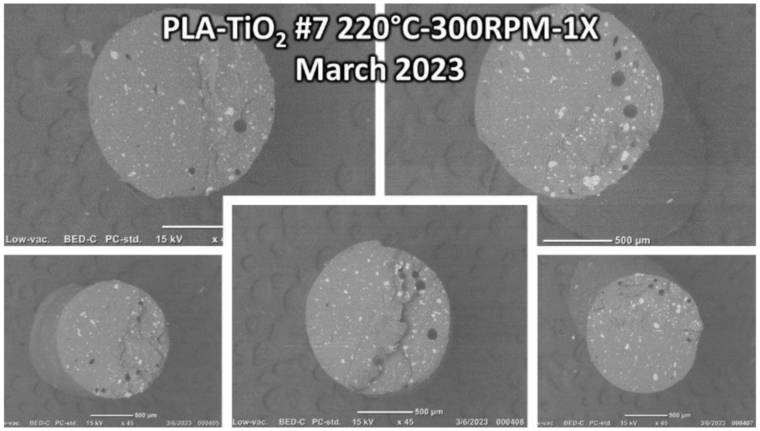

H.

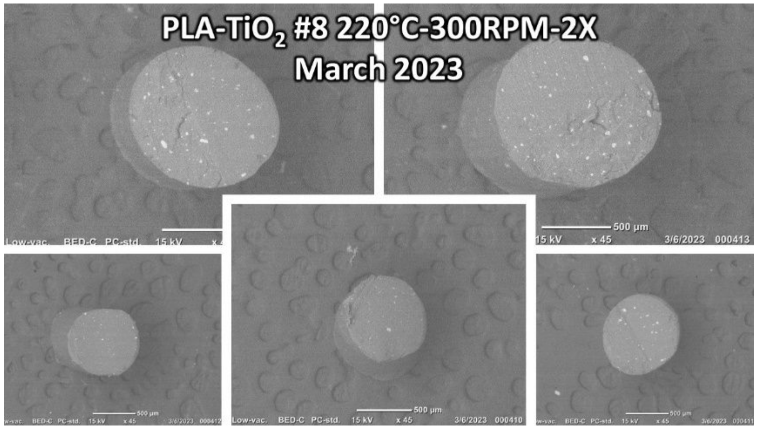

Supporting Figure S6. SEM characterization of filament cross sections. A-H represent treatments 1-8.

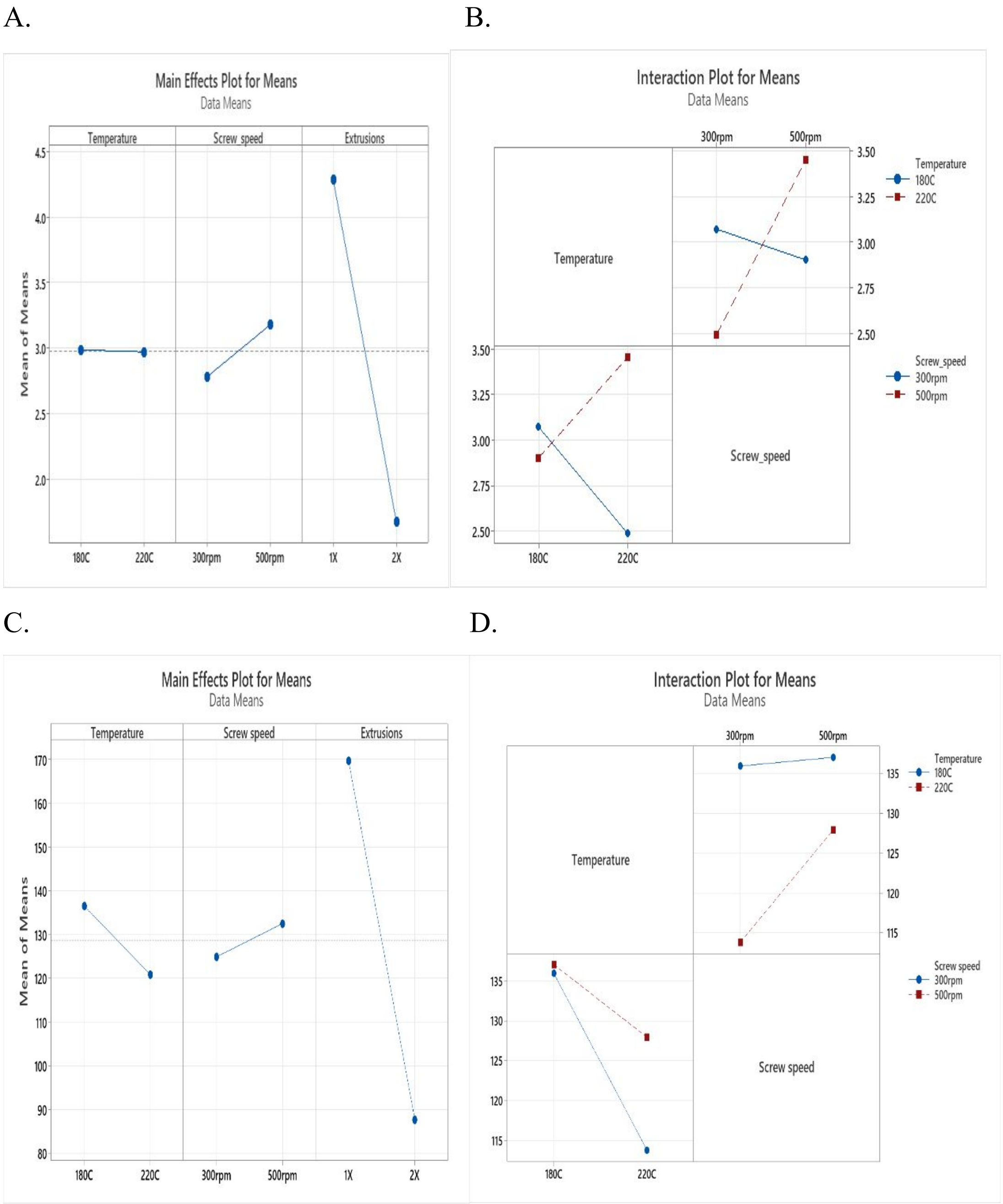

Supporting Figure S7. Results of the Taguchi Design of Experiments of the eight different processing treatments on total agglomerate area (Panels A,B) and number of agglomerates (Panels C,D). Panels B and D show interactions between the factors extrusion temperature and screw speed.

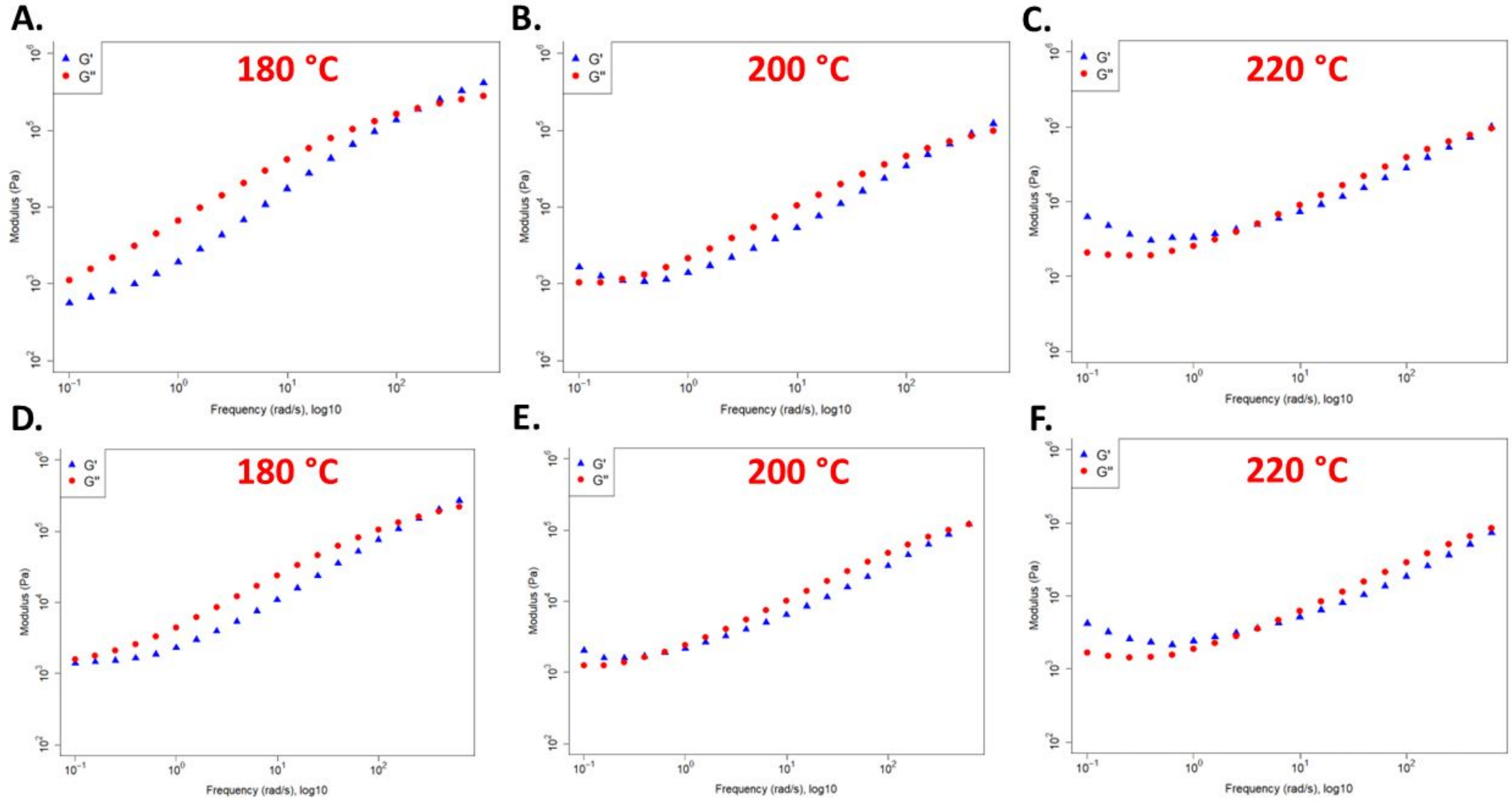

Supporting Figure S8. Moduli of the PLA-TiO_2 composite filaments at different relevant processing temperatures. Panels A-C summarize Treatment #3 and Panels D-F summarize Treatment #4. The test temperatures were 180 °C (Panels A,D), 200 °C (Panels B,E) and 220 °C (Panels C,F)

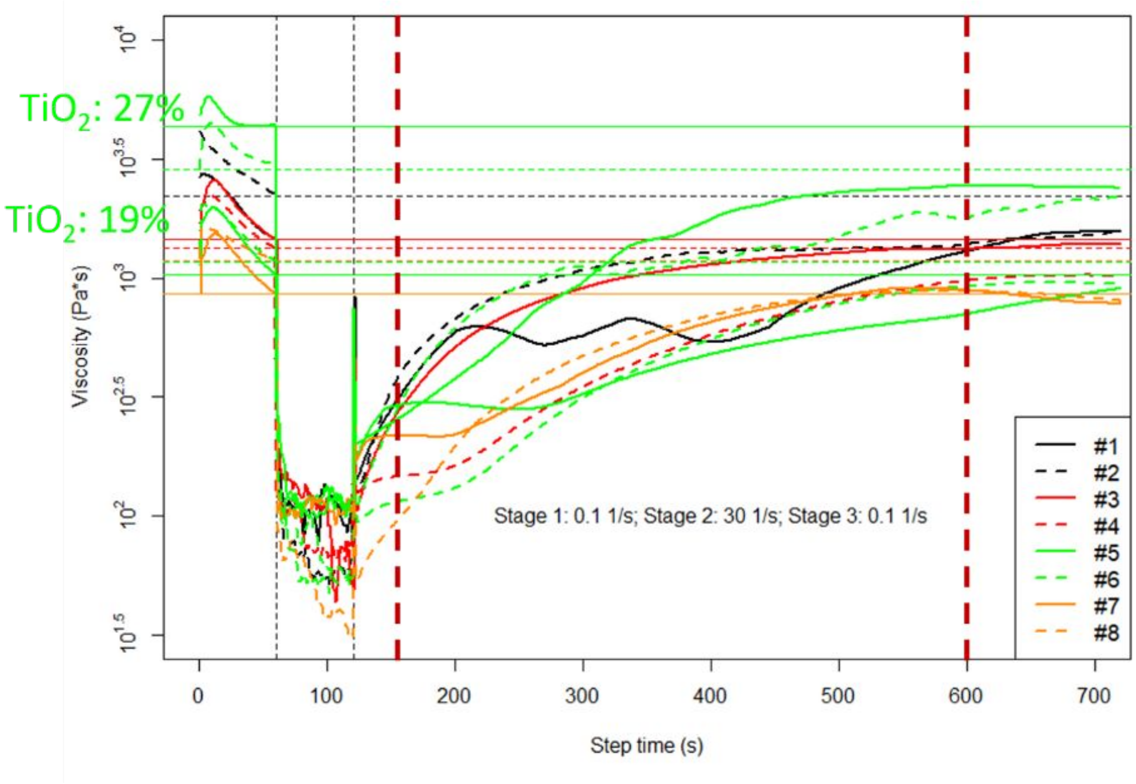

Supporting Figure S9. Three interval thixotropy tests (3ITT) to determine recovery in viscosity conducted in steady shear at 200 °C. The 3ITT viscosity at low shear (0.1 s^{-1}) followed by higher shear (30 s^{-1}) followed by a recovery period at low shear (0.1 s^{-1}).

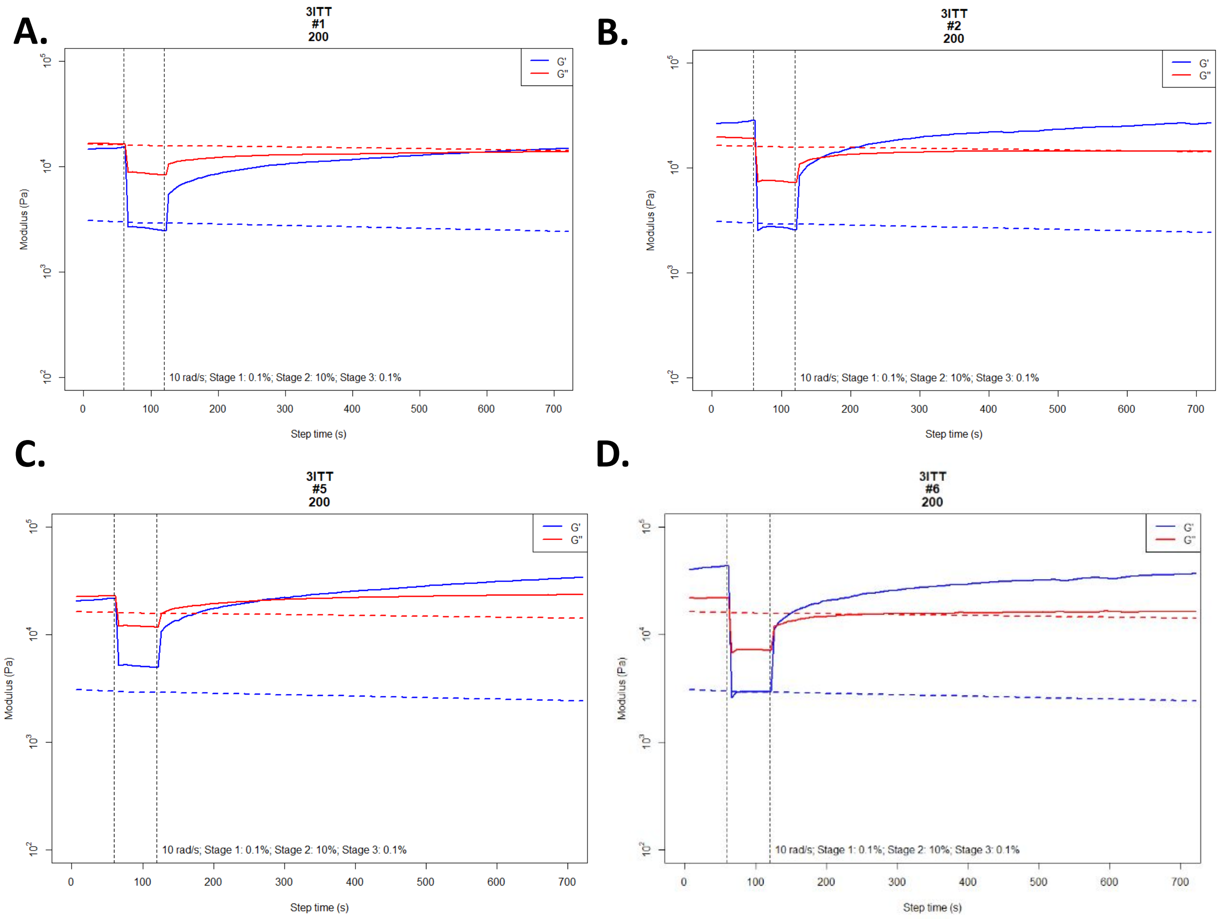

Supporting Figure S10. Modulus recovery experiments for the composite filaments processed at 180 ºC conducted in oscillation (10 rad/s). Panels summarize data for different treatments (TRMT): (A) TRMT#1 (180ºC; 300 rpm; 1X extrusion); (B) TRMT#2 (180ºC; 300 rpm; 2X extrusions); (C) TRMT#5 (180ºC; 500 rpm; 1X extrusion); (D) TRMT#6 (180ºC; 500 rpm; 2X extrusions). Interval 1 was low strain within LVR (0.1%), interval 2 was high strain outside LVR (10%) and interval 3 was low strain within LVR (0.1%) for modulus recovery. Dashed lines represent neat PLA behavior.

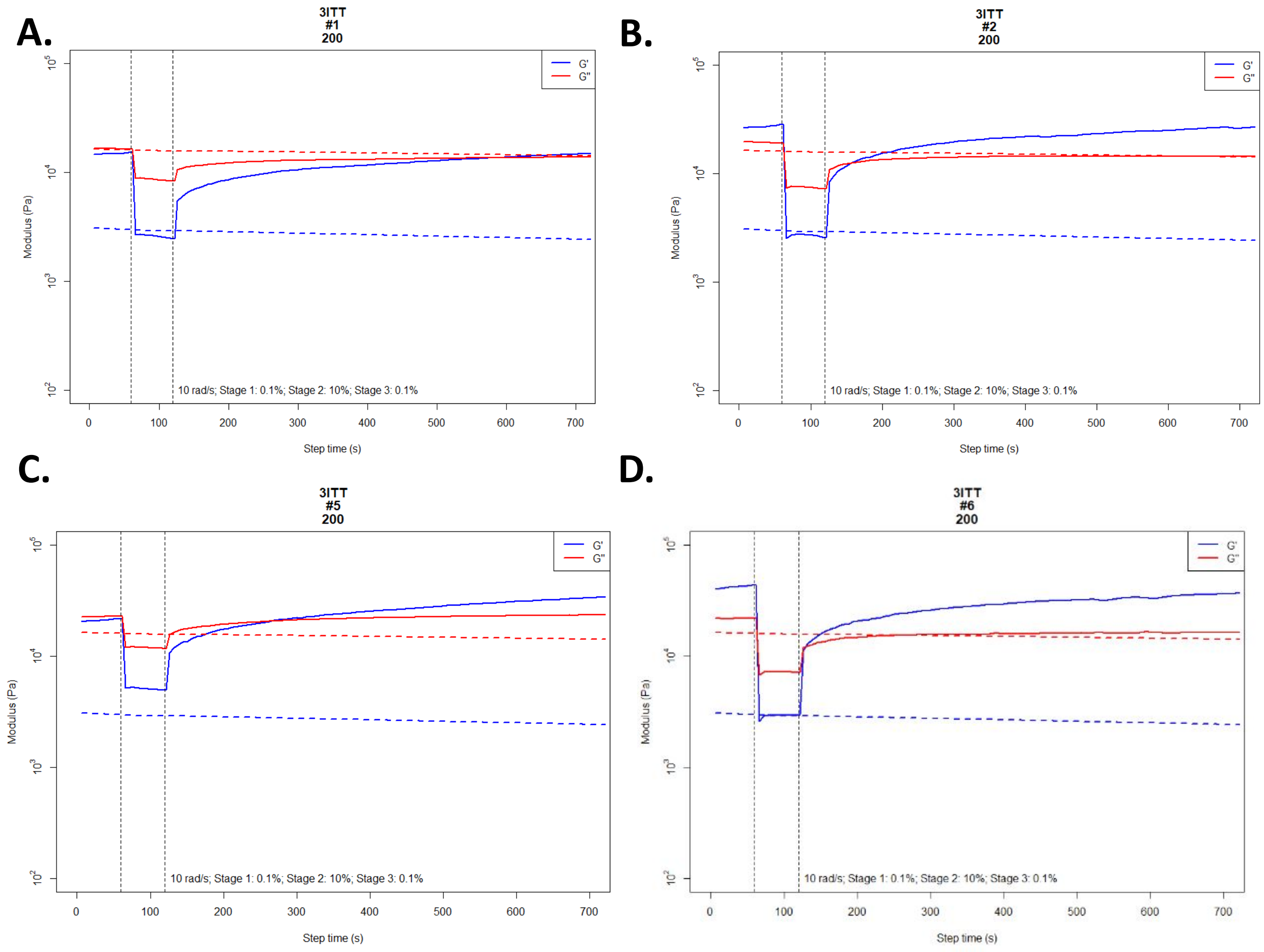

Supporting Figure S11. Modulus recovery experiments for the composite filaments processed at 220 °C conducted in oscillation (10 rad/s). Panels summarize data for different treatments (TRMT): (A) TRMT#1 (180ºC; 300 rpm; 1X extrusion); (B) TRMT#2 (180ºC; 300 rpm; 2X extrusions); (C) TRMT#5 (180ºC; 500 rpm; 1X extrusion); (D) TRMT#6 (180ºC; 500 rpm; 2X extrusions). Interval 1 was low strain within LVR (0.1%), interval 2 was high strain outside LVR (10%) and interval 3 was low strain within LVR (0.1%) for modulus recovery. Dashed lines represent neat PLA behavior.

A.

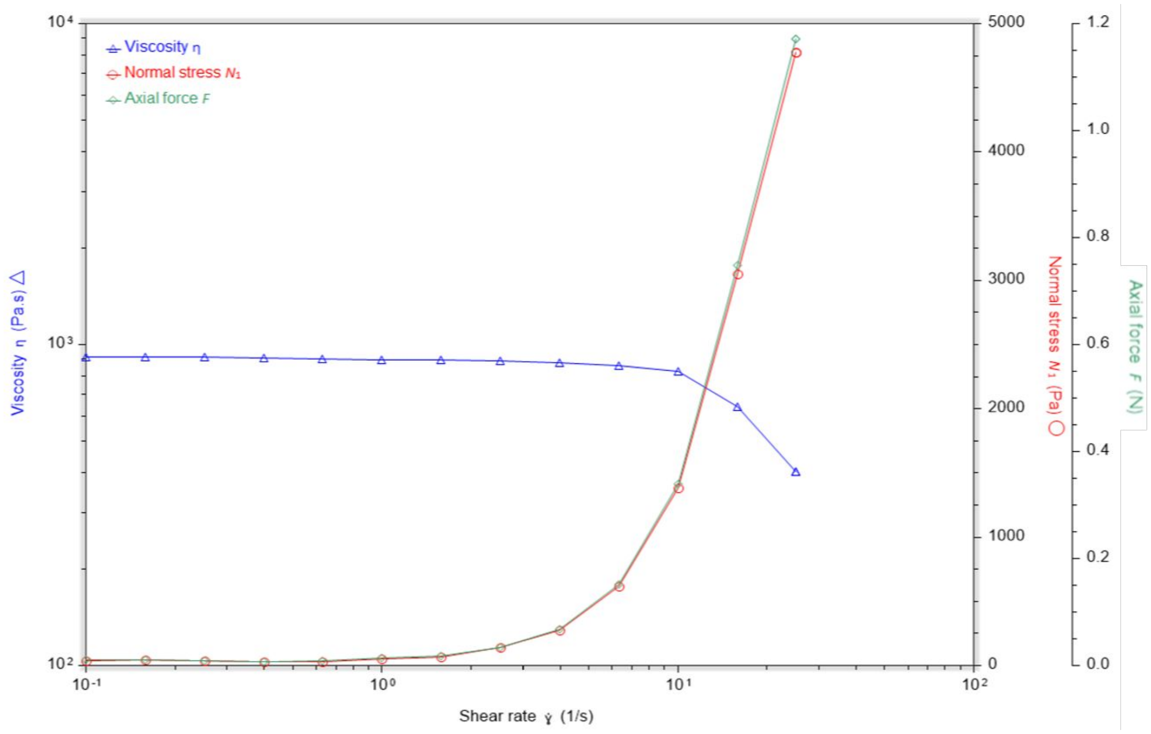

B.

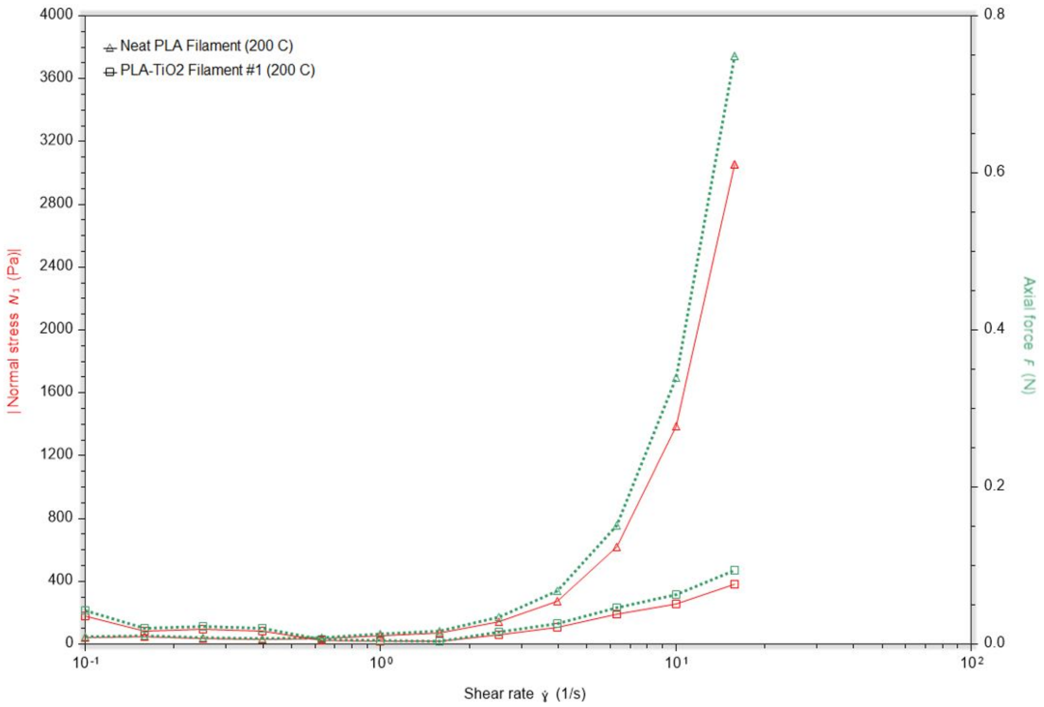

Supporting Figure S12. Shear sweeps conducted at 200 °C; (A) viscosity, normal stress, axial force and viscosity for neat PLA vs. shear rate; and (B) normal stress and axial force vs. shear rate for neat PLA and PLA-TiO_2.

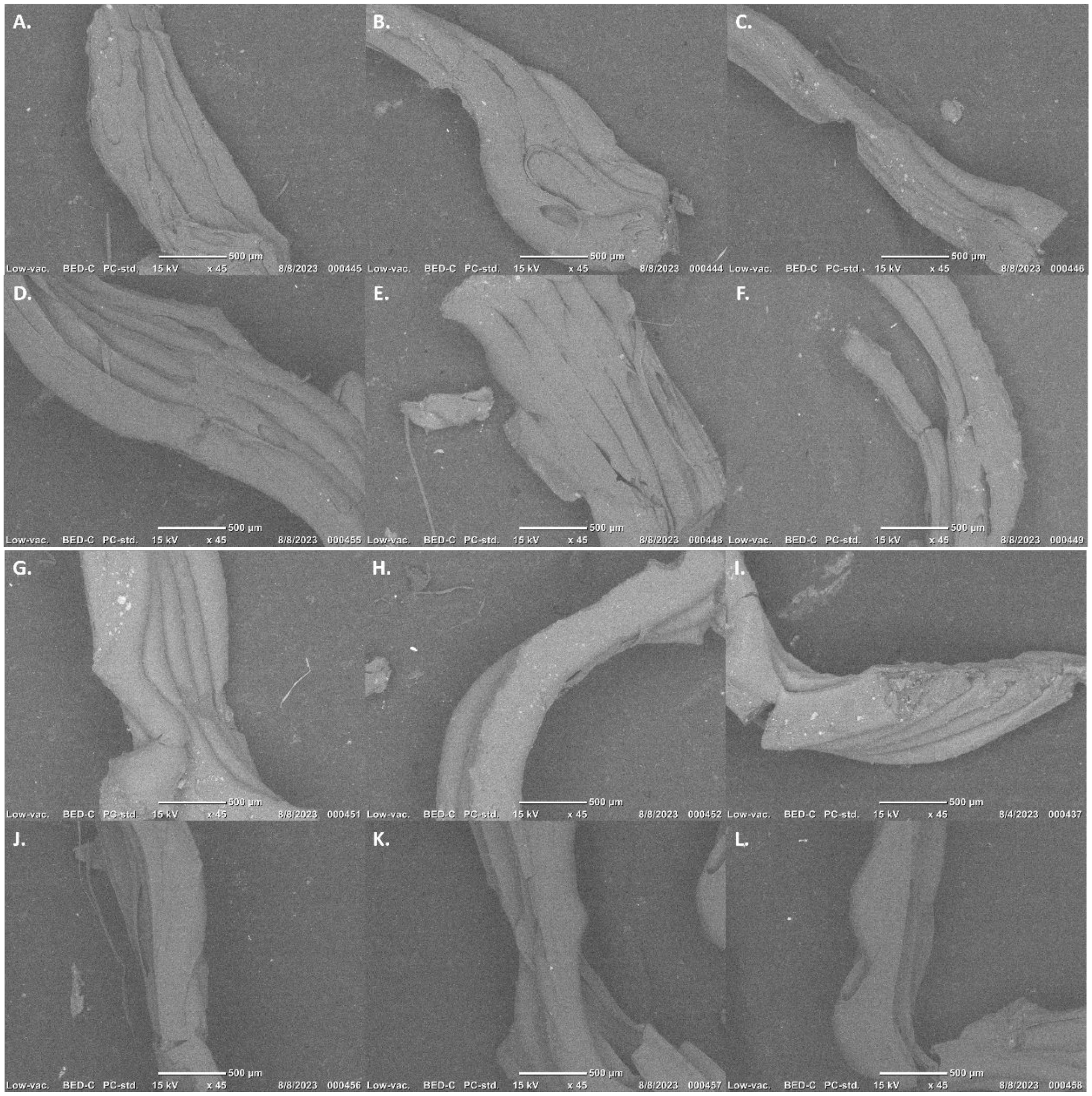

Supporting Figure S13. Scanning electron microscopy images of the printed gyroid infill of the test disks. The panels summarize treatment #1 (A-C), treatment #4 (D-F), treatment #5 (G-I) and treatment #6 (J-L).

Degradation at the relevant processing temperature of 220 °C for both neat PLA and PLA-TiO_2 (20% w/w TiO_2) were determined using a TGA (TA Instruments, TA-5500-0510) by placing 10-14 mg samples on platinum pans. The "heat and hold function" was selected and the temperature was ramped from 20 to 220 °C at a rate of 50 °C/min in air and then held at 220 °C for 90 minutes to monitor weight loss. Results (Supporting Figure S-14) indicate minor weight loss, with slightly greater weight loss observed for the PLA-TiO_2.

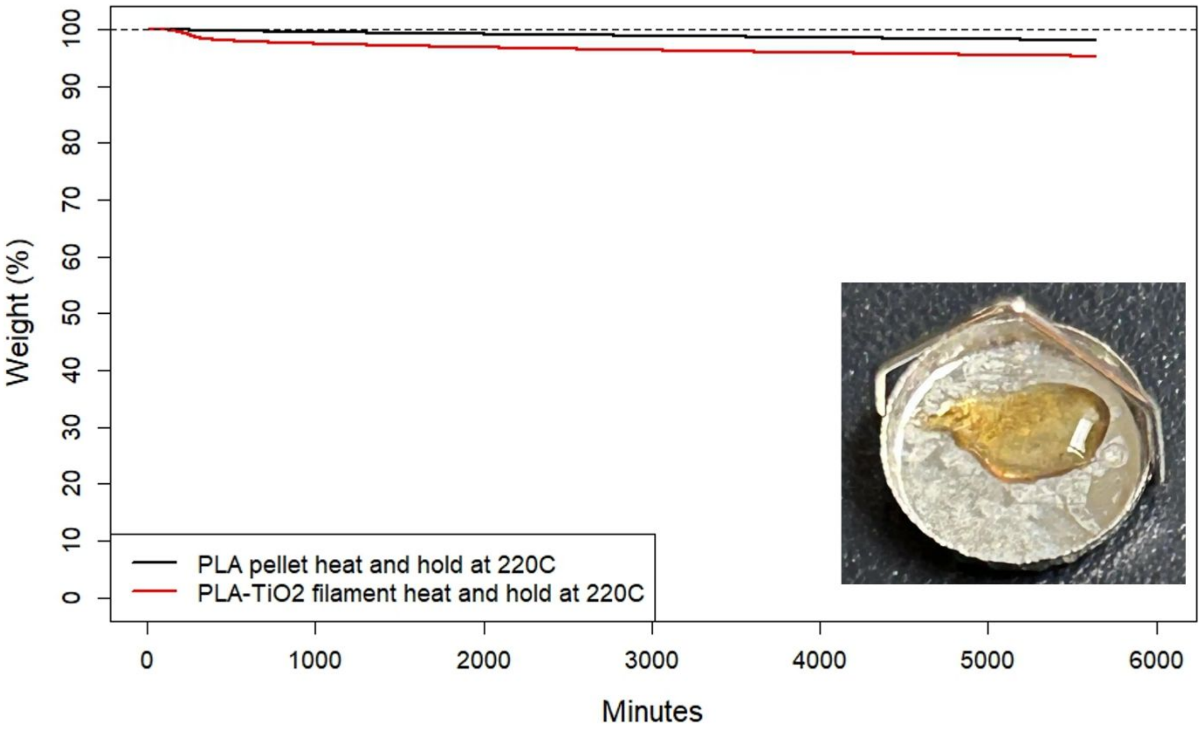

Supporting Figure S14. Thermal Gravimetric Analysis of neat polylactic acid (PLA) and 20% w/w TiO_2 integrated into PLA pellets. The inset shows the slight browning in coloration of the PLA after heating.

References

(1) Papageorgiou, G. Z.; Karandrea, E.; Giliopoulos, D.; Papageorgiou, D. G.; Ladavos, A.; Katerinopoulou, A.; Achilias, D. S.; Triantafyllidis, K. S.; Bikiaris, D. N. Effect of clay structure and type of organomodifier on the thermal properties of poly(ethylene terephthalate) based nanocomposites. *Thermochimica Acta* **2014**, *576*, 84-96.
(2) Yasuniwa, M.; Sakamo, K.; Ono, Y.; Kawahara, W. Melting behavior of poly(l-lactic acid): X-ray and DSC analyses of the melting process. *Polymer* **2008**, *49* (7), 1943-1951.
(3) Fukushima, K.; Abbate, C.; Tabuani, D.; Gennari, M.; Camino, G. Biodegradation of poly(lactic acid) and its nanocomposites. *Polymer Degradation and Stability* **2009**, *94* (10), 1646-1655.
(4) Fonseca, J.; Ferreira, I. A.; de Moura, M. F. S. F.; Machado, M.; Alves, J. L. Study of the interlaminar fracture under mode I loading on FFF printed parts. *Composite Structures* **2019**, *214*, 316-324.
(5) Fonseca, C.; Ochoa, A.; Ulloa, M. T.; Alvarez, E.; Canales, D.; Zapata, P. A. Poly(lactic acid)/TiO2 nanocomposites as alternative biocidal and antifungal materials. *Materials Science and Engineering: C* **2015**, *57*, 314-320.
(6) Villmow, T.; Kretzschmar, B.; Pötschke, P. Influence of screw configuration, residence time, and specific mechanical energy in twin-screw extrusion of polycaprolactone/multi-walled carbon nanotube composites. *Composites Science and Technology* **2010**, *70* (14), 2045-2055.
(7) Rueda, M. M.; Auscher, M.-C.; Fulchiron, R.; Perie, T.; Martin, G.; Sonntag, P.; Cassagnau, P. Rheology and applications of highly filled polymers: A review of current understanding. *Progress in Polymer Science* **2017**, *66*, 22-53.
